# Programming multicellular assembly with synthetic cell adhesion molecules

**DOI:** 10.1038/s41586-022-05622-z

**Published:** 2022-12-12

**Authors:** Adam J. Stevens, Andrew R. Harris, Josiah Gerdts, Ki H. Kim, Coralie Trentesaux, Jonathan T. Ramirez, Wesley L. McKeithan, Faranak Fattahi, Ophir D. Klein, Daniel A. Fletcher, Wendell A. Lim

**Affiliations:** 1 UCSF Cell Design Institute, University of California, San Francisco, CA USA; 2 Department of Cellular and Molecular Pharmacology, University of California, San Francisco, CA USA; 3 Center for Cellular Construction, University of California, San Francisco, CA USA; 4 Department of Bioengineering, University of California, Berkeley, CA USA; 5 Department of Neurology, Weill Institute for Neuroscience, University of California, San Francisco, CA USA; 6 Program in Craniofacial Biology, University of California, San Francisco, CA USA; 7 Department of Orofacial Sciences, University of California, San Francisco, CA USA; 8 Eli and Edythe Broad Center of Regeneration Medicine and Stem Cell Research, University of California, San Francisco, CA USA; 9 Department of Pediatrics, Cedars-Sinai Medical Center, Los Angeles, CA USA; 10 Chan Zuckerberg Biohub, San Francisco, CA USA; 11 Present Address: Department of Mechanical and Aerospace Engineering, Carleton University, Ottawa, Ontario Canada; 12 Present Address: Maze Therapeutics, San Francisco, CA USA

**Keywords:** Synthetic biology, Cadherins, Protein engineering, Integrins, Tissue engineering

## Abstract

Cell adhesion molecules are ubiquitous in multicellular organisms, specifying precise cell–cell interactions in processes as diverse as tissue development, immune cell trafficking and the wiring of the nervous system^1–4^. Here we show that a wide array of synthetic cell adhesion molecules can be generated by combining orthogonal extracellular interactions with intracellular domains from native adhesion molecules, such as cadherins and integrins. The resulting molecules yield customized cell–cell interactions with adhesion properties that are similar to native interactions. The identity of the intracellular domain of the synthetic cell adhesion molecules specifies interface morphology and mechanics, whereas diverse homotypic or heterotypic extracellular interaction domains independently specify the connectivity between cells. This toolkit of orthogonal adhesion molecules enables the rationally programmed assembly of multicellular architectures, as well as systematic remodelling of native tissues. The modularity of synthetic cell adhesion molecules provides fundamental insights into how distinct classes of cell–cell interfaces may have evolved. Overall, these tools offer powerful abilities for cell and tissue engineering and for systematically studying multicellular organization.

## Abstract (web summary)

Synthetic cell adhesion molecules yield customized cell–cell interactions with adhesion properties that are similar to native interactions, and offer abilities for cell and tissue engineering and for systematically studying multicellular organization.

## Main

**Fig. 1:**
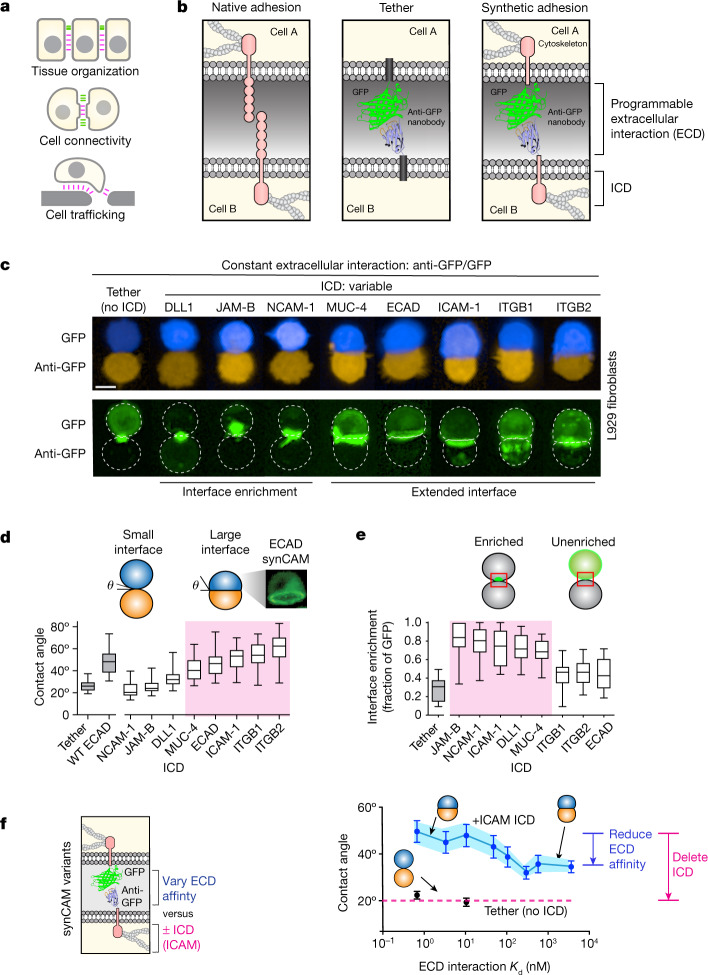
synCAMs facilitate custom cell–cell interactions. **a**, Diverse functional roles of cell adhesion. **b**, The conceptual design of synCAM receptors. The extracellular domain of a CAM (left) is replaced by GFP and a GFP-binding nanobody (anti-GFP; right). A tether control lacking an ICD is also shown (middle). **c**, Maximum projection of ×20 confocal microscopy images of pairwise synCAM interfaces. Scale bar, 10 µm. *t* = 3 h. A GFP-expressing cell (blue) is bound to an anti-GFP-expressing cell (orange). The CAM TM and ICD domain for each pair is indicated (tether is the control lacking the ICD) (top). Bottom, the GFP channel of the interfaces above, highlighting the differences in receptor enrichment at the interface. Matched synCAM expression levels are shown in Extended Data Fig. 1. **d**, The contact angles measured from the interfaces shown in **a**. *n* = 20 (tether), *n* = 20 (WT ECAD), *n* = 20 (DLL1), *n* = 20 (JAM-B), *n* = 20 (NCAM-1), *n* = 20 (ICAM-1), *n* = 20 (ECAD), *n* = 20 (ITGB1), *n* = 20 (ITGB2), *n* = 15 (MUC4). Contact angles for WT ECAD homotypic cell–cell interaction are also shown. **e**, The fraction of GFP enrichment at the cell–cell interface from **c**. *n* = 20 (tether), *n* = 20 (DLL1), *n* = 20 (JAM-B), *n* = 20 (NCAM-1), *n* = 20 (ICAM-1), *n* = 20 (ECAD), *n* = 20 (ITGB1), *n* = 20 (ITGB2), *n* = 15 (MUC4). **f**, Quantification of the contact angles of pairwise L929 cells expressing GFP/anti-GFP synCAMs with the indicated affinities and in the presence (blue) or absence (black) of an ICAM-1 ICD. *n* = 20 pairs. The error bars show the 95% confidence intervals. *t* = 3 h. Matched synCAM expression levels are shown in Extended Data Fig. 1. An alternative analysis (competition cell sorting assay) of the same series of altered affinity synCAM cells is shown in Extended Data Fig. 3. For the box plots in **d** and **e**, the centre line shows the median, the box limits show the 25th to 75th percentile and the whiskers show the minimum to maximum values. Source data

The ability to systematically program cell–cell adhesion would provide powerful new tools to study development, neurobiology and immunology, and could facilitate the repair of multicellular tissues and the design of therapeutic cells^5,6^ (Fig. 1a). Nonetheless, engineering adhesion in metazoan cells remains an underexplored area in synthetic biology.

Native cell–cell interactions are mediated by a large collection of cell adhesion molecules (CAMs)—complex transmembrane proteins that bind neighbouring cells or matrix and induce a mechanical adhesive response, often involving cytoskeletal rearrangements^7–11^. Examples of CAMs include integrins, which assemble focal adhesions, and cadherins, which assemble adherens junctions between epithelial cells^11–14^. The structural complexity and functional diversity of CAMs make it unclear whether the extracellular binding and intracellular-domain-mediated cytoskeletal reorganization functions can be uncoupled and recombined to generate new cell–cell connectivities, although previous studies indicate the potential for modularity^15–19^.

Here we systematically explore the modularity of CAMs by fusing orthogonal extracellular binding domains (ECDs) to endogenous CAM intracellular domains (ICDs), thereby generating synthetic CAMs (synCAMs). We characterize the resulting cell–cell interfaces, and test whether synCAMs can program new multicellular organization.

## Synthetic CAMs show native-like adhesion

We generated heterophilic synCAMs in which a well-characterized orthogonal binding interaction—the GFP–anti-GFP (nanobody) interaction—is fused to the ICDs of E-cadherin (ECAD), integrin β1 (ITGB1), integrin β2 (ITGB2), intercellular adhesion molecule 1 (ICAM-1), delta-like protein 1 (DLL1), junctional adhesion molecule B (JAM-B), neural cell adhesion molecule 1 (NCAM-1) and mucin 4 (MUC-4)^20^ (Fig. 1b). The transmembrane region (TM) and ICD from the donor CAM was fused to the GFP or anti-GFP ECD.

**Extended Data Fig. 1:**
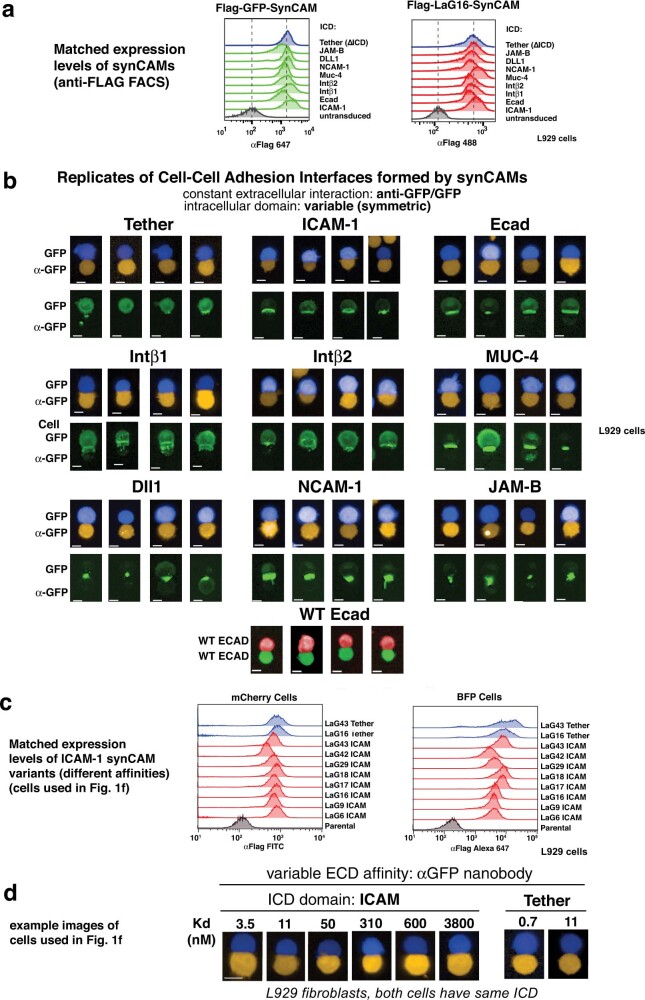
Characterization of synCAM expression and function in L929 fibroblast cells (*linked to main* Fig. 1c–f). (**a**) FACS analysis of GFP (left) and αGFP (right) synCAM expression in L929 fibroblast cells following cell sorting. Surface expression of each synCAM is measured using labelled anti-FLAG tag antibody. The CAM TM and ICD domain for each construct is indicated (tether = control lacking ICD, DLL1 = Delta-like Protein 1, JAM-B = Junction Adhesion Molecule B, NCAM-1 = Neural Cell Adhesion molecule 1, MUC-4 = Mucin 4, ICAM-1 = Intercellular Adhesion Molecule 1, Ecad = E-cadherin, Intβ1 = beta 1 integrin, Intβ2 = beta 2 integrin). Analysis shows that surface expression levels of the tether and alternative synCAM constructs are well matched. (**b**) Additional replicates of synCAM cell–cell adhesion interface analysis. Maximum projection of 20x confocal microscopy images of pairwise synCAM interfaces (t = 3 h): GFP-expressing cell (blue) is bound to an αGFP expressing cell (orange). The GFP channel of the interfaces is shown, highlighting differences of receptor enrichment. Four out of twenty additional examples are shown here. (**c**) FACS analysis of αGFP synCAM expression in L929 fibroblast cells expressing cytosolic mCherry (left) or BFP (right) following cell sorting. The CAM TM and ICD domain for each construct is ICAM-1, and the GFP-binding llama nanobody (LaG) ECD for each construct is indicated. This analysis shows that this series of alternative affinity synCAMs are expressed at comparable levels. (**d**) Maximum projection of 20X confocal microscopy images of pairwise synCAM interfaces (t = 3 h): GFP-expressing cell (blue) is bound to an αGFP expressing cell with the indicated binding *K*_d_ (orange).

We then tested whether cognate synCAM paired with symmetrically matched ICDs can drive junction formation between L929 mouse fibroblasts (a cell line with low endogenous adhesion that is used to assess cadherin differential adhesion sorting)^21,22^. Cells expressing cognate synCAMs were mixed in a flat-bottom, ultra-low-attachment (ULA) plate, and imaged using confocal microscopy (Fig. 1c). We compared synCAM-driven interfaces with those formed by native adhesion molecules (for example, wild-type (WT) ECAD) or by a simple tether (GFP or anti-GFP fused to a transmembrane domain lacking any ICD). synCAMs/tethers were expression matched (Extended Data Fig. 1).

Several synCAMs (ICDs: ECAD, ITGB1, ITGB2, ICAM-1 and MUC-4) formed extensive interfaces comparable to those observed with native cadherin. Native-like interfaces form despite these molecules completely lacking their large native extracellular domains. By contrast, the tether (no ICD) did not form an extensive interface, showing only a small point of contact.

Several other synCAMs (ICDs: NCAM-1, JAM-B and DLL1) exhibited a distinct phenotype. The resulting interfaces were small, but with substantial interface enrichment of the GFP-labelled synCAMs (Fig. 1c). By contrast, the GFP signal in tethered cell pairs remained distributed throughout the entire membrane. Thus, these synCAMs drive a distinct phenotype of enriched spatial clustering at the interface when engaged.

To quantitatively analyse interface geometry for synCAM interactions (15–20 cell pairs), we measured contact angle—a standard metric of apparent cell–cell surface tension that is correlated with interface size^23–25^ (Fig. 1d). We also measured the enrichment fraction (the fraction of GFP-tagged synCAM localized to the interface versus total membrane; Fig. 1e). These results show two main phenotypic classes of synCAMs: one class induces the formation of large, extensive cell–cell interfaces (ICDs: ECAD, ITGB1, ITGB2, and ICAM-1, MUC-4), and another class induces the formation of small but highly enriched interfaces (ICDs: NCAM-1, JAM-B and DLL1) (MUC-4 and ICAM-1 show hybrid behaviours). Each of these synCAM interface classes is distinct from the simple tether interaction.

## The ICD determines interface strength

**Extended Data Fig. 2:**
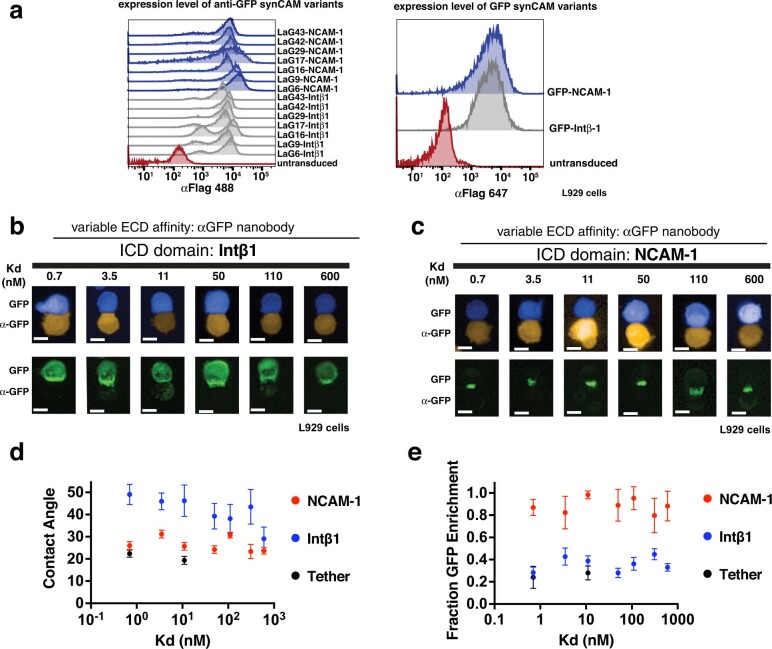
Changing ECD affinity has minor effect on function of multiple synCAMs: Intβ1 and NCAM ICDs (linked to Fig. 1f). (**a**) FACS analysis of αGFP synCAM expression in L929 fibroblast cells expressing cytosolic mCherry following cell sorting. The CAM TM and ICD domain for each construct is NCAM-1 or Intβ1, and the GFP-binding llama nanobody (LaG) ECD for each construct is indicated. This analysis shows that this series of alternative affinity synCAMs are expressed at comparable levels. (**b**) Maximum projection of 20X confocal microscopy images of pairwise synCAM interfaces (t = 3 h, scale bar = 10 µm). Top: GFP-expressing cell (blue) is bound to an αGFP expressing cell (orange). The CAM TM and ICD domain for each pair is Intβ1. Bottom: GFP channel of the interfaces above highlighting differences of receptor enrichment at the interface. (**c**) Maximum projection of 20X confocal microscopy images of pairwise synCAM interfaces (t = 3 h, scale bar = 10 µm). Top: GFP-expressing cell (blue) is bound to an αGFP expressing cell (orange). The CAM TM and ICD domain for each pair is NCAM-1. Bottom: GFP channel of the interfaces above highlighting differences of receptor enrichment at the interface. (**d**) Plots of contact angles measured from the interfaces shown in b and c in relation to the corresponding LaG nanobody affinity (data are presented as mean values of of n = 10 pairs, error = 95 % CI). The contact angles for Intβ1 (blue) are shown in relation to NCAM-1 (red) and the tether control from Fig. 1f (black). (**e**) Plots of GFP enrichment measured from the interfaces shown in b and c in relation to the corresponding LaG nanobody affinity (data are presented as mean values of n = 10 pairs, error = 95 % CI). The GFP enrichment for NCAM-1 (red) are shown compared to Intβ1 (blue) and the tether control from Fig. 1f (black). Source data

Both a strong ECD binding interaction and strong ICD coupling with the cytoskeleton could contribute to tight cell–cell interface formation. The synCAM modularity enables investigation of the relative ECD and ICD contributions to interface strength. Using the ICAM-1 synCAM as a testbed system, we characterized cell–cell interfaces with varied ECD affinity (using an affinity series of GFP nanobodies) or a deleted ICD^20^ (Fig. 1f and Extended Data Fig. 1). Reducing the ECD affinity from a dissociation constant (*K*_d_) of 0.7 nM to 3 µM (>10^3^ fold) gradually decreases the resulting cell–cell contact angle, but even the weakest ECD exhibits a significantly expanded interface. By contrast, deletion of the ICAM-1 ICD, even in the presence of a high-affinity ECD, disrupts the interface completely. A similar modest decrease in cell–cell contact angle was observed for synCAMs with an ITGB1 ICD when the ECD *K*_d_ was varied between 0.7 nM to 110 nM (Extended Data Fig. 2). These observations are consistent with a model in which cytomechanical changes mediated by the ICDs have a dominant role in determining the interface strength and morphology^23,24^.

We also characterized how decreasing the ECD interaction affinity affects the interface enrichment phenotype of the NCAM-1 synCAM. The GFP receptor remains highly enriched at the interface even when the ECD affinity is varied over a range of *K*_d_ = 0.7 nM to 600 nM (Extended Data Fig. 2). Thus, the enriched interface phenotype also appears to be driven largely by the ICD identity.

**Extended Data Fig. 3:**
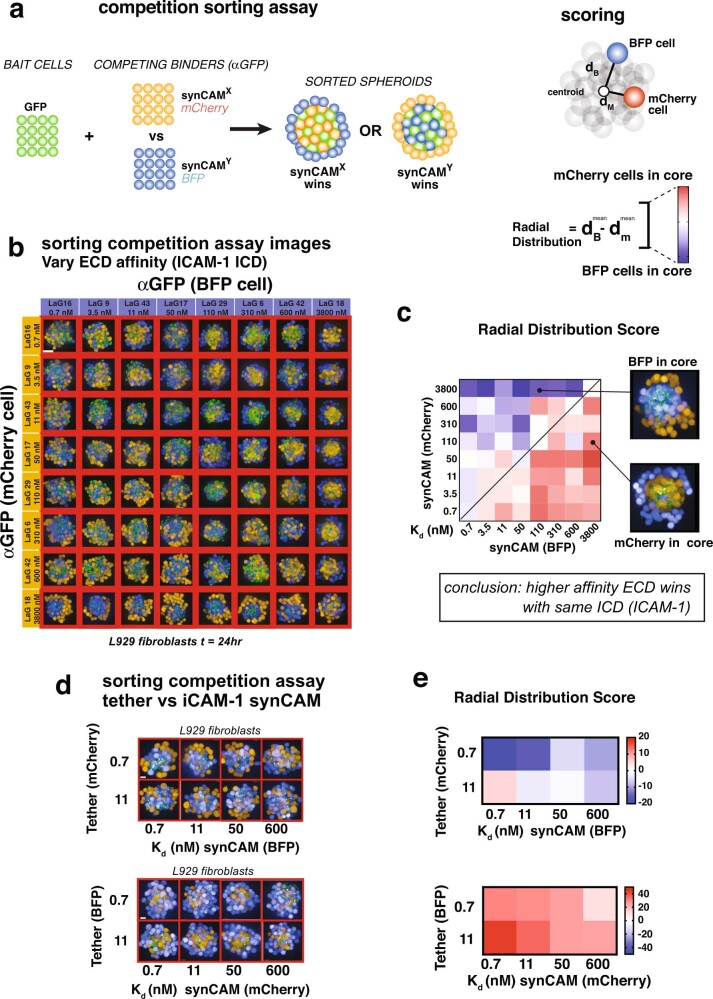
Differential sorting of synCAMs with varying ECD affinity and ICD (*linked to main* Fig. 1f). (**a**) Cartoon depiction of the differential sorting competition assay (left) and quantification of radial distribution that is represented as a heat map (right). This experiment represents an alternative way to measure adhesion preferences/strength of the diverse synCAM-driven cell–cell interactions that differs from the contact angle measurement shown in Fig. 1f. Here we mix surface GFP L929 cells (bait cells) with two competing differentially labelled L929 cells, each with a different αGFP synCAM. Stronger adhesion of the synCAM is assessed via the relative degree of co-sorting of the competitor cells to the core in conjunction with the bait cells. We calculate the radial distribution of competing cells (red/blue) from the centroid of the spheroid. (**b**) Representative maximum projection images of cell sorting competition assay between L929 cells expressing αGFP-ICAM-1 with the indicated ECD LaG nanobody (mCherry or BFP) mixed with L929 cells expressing GFP-ICAM-1 (t = 24 h, scale bar = 50 µm). (**c**) Quantification of the cell sorting competition assay from b (n = 4). (**d**) Representative maximum projection images of cell sorting competition assay between L929 cells expressing αGFP-ICAM-1 and cytosolic and L929 cells expressing αGFP-Tether mixed with L929 cells expressing GFP-ICAM-1 (scale bar = 20 µm, t = 24 h). (**e**) Quantification of the cell sorting competition assay from d (n = 4). Source data

**Extended Data Fig. 4:**
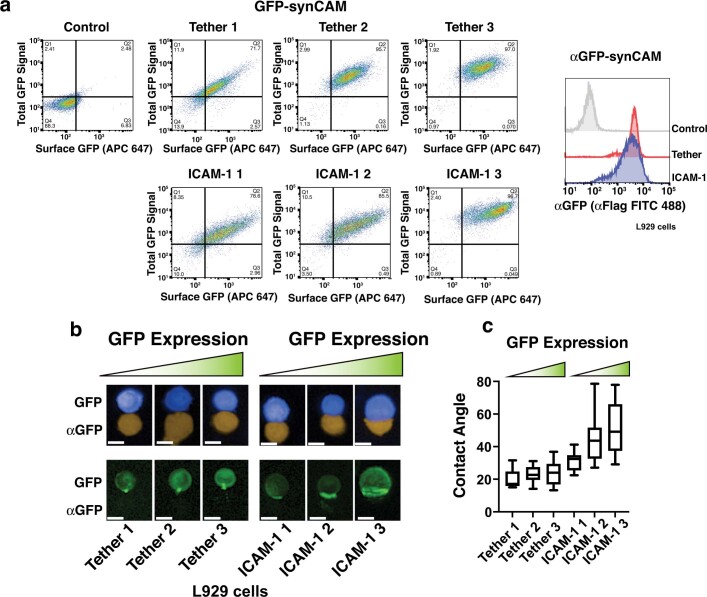
Characterization of tuning synCAM expression. (**a**) FACS analysis of GFP synCAM and αGFP synCAM expression in L929 fibroblast cells following cell sorting with an ICAM-1 or Tether ICD. For the GFP constructs, expression is shown both for total GFP signal in the cell (Y-axis) and cells stained with an αFlag APC 647 antibody (x-axis). (**b**) Maximum projection of 20X confocal microscopy images of pairwise synCAM interfaces (t = 3 h, scale bar = 10 µm) of different expression levels from panel a: GFP-expressing cell (blue) is bound to an αGFP expressing cell (orange). The CAM TM and ICD domain for each pair is ICAM-1 or Tether. (**c**) Box and whisker plots (box = 25th to 75th percentile, whiskers = min to max, centre = median) of contact angles measured from the interfaces shown in b (n = 10 pairs). Source data

The dominance of the ICD over ECD affinity in determining adhesion properties was corroborated in competition sorting assays (Extended Data Fig. 3). Here, cells expressing two different anti-GFP synCAM (ICAM-1 ICD) variants compete to co-sort with GFP synCAM ‘bait’ cells. Higher-affinity cells preferentially sort with the bait cells to the core of the cell cluster. This complementary assay also indicates that the ICD primarily determines adhesion preferences. Expression of GFP–ICAM-1/anti-GFP–ICAM-1 at higher levels also increased the contact angle (Extended Data Fig. 4). By contrast, higher expression of the GFP/anti-GFP tethers did not change contact angle.

## Two classes of interface morphologies

**Fig. 2:**
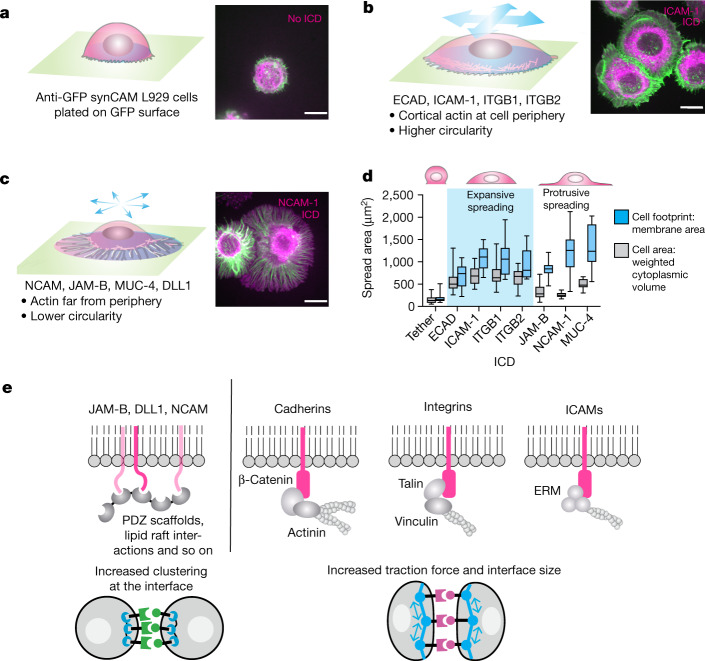
SynCAM ICDs yield distinct mechanical and morphological properties. **a**–**c**, Representative Phalloidin-stained images of L929 cells expressing the indicated synCAMs spreading on a GFP-coated surface. Scale bars, 10 µm. *t* = 2 h. Actin (Phalloidin stain) is shown in green; the full footprint of the cell (membrane label) is shown in purple. All of the images are shown at the same scale. **a**, The L929 cell expressing anti-GFP–tether (no ICD) shows minimal spreading. **b**, L929 cells expressing synCAMs with ICDs from ECAD, ICAM-1, ITGB1 and ITGB2 show an expansive spreading phenotype—the cell spreads in circular manner with cortical actin at the periphery of the cell footprint. See the spreading kinetic assays in Extended Data Fig. 5. **c**, L929 cells expressing synCAMs with ICDs from NCAM-1, JAM-B and MUC-4 show a protrusive spreading phenotype (a ‘fried egg’ shape)—cortical actin does not spread very far, but the cell membrane footprint extends in a very thin layer beyond the cell, often with less circularity (that is, more filopodial or lamellopodial in nature). **d**, The full footprint of the cell (blue) and cell area (grey) for synCAM-mediated cell spreading. For the box plots, the centre line shows the median, the box limits show the 25th to 75th percentile and the whiskers show the minimum to maximum values. Cell area: *n* = 23 (tether), *n* = 17 (ECAD), *n* = 23 (JAM-B), *n* = 16 (ICAM-1), *n* = 16 (ITGB1), *n* = 18 (ITGB2), *n* = 14 (NCAM-1), *n* = 12 (MUC-4). Cell footprint: *n* = 22 (tether), *n* = 21 (ECAD), *n* = 19 (JAM-B), *n* = 23 (ICAM-1), *n* = 16 (ITGB1), *n* = 12 (ITGB2), *n* = 14 (NCAM-1), *n* = 15 (MUC-4). **e**, Known recruitment interactions of downstream intracellular proteins found in CAM ICDs. See the mutational analysis of ICD-binding motifs in Extended Data Fig.6. Source data

**Extended Data Fig. 5:**
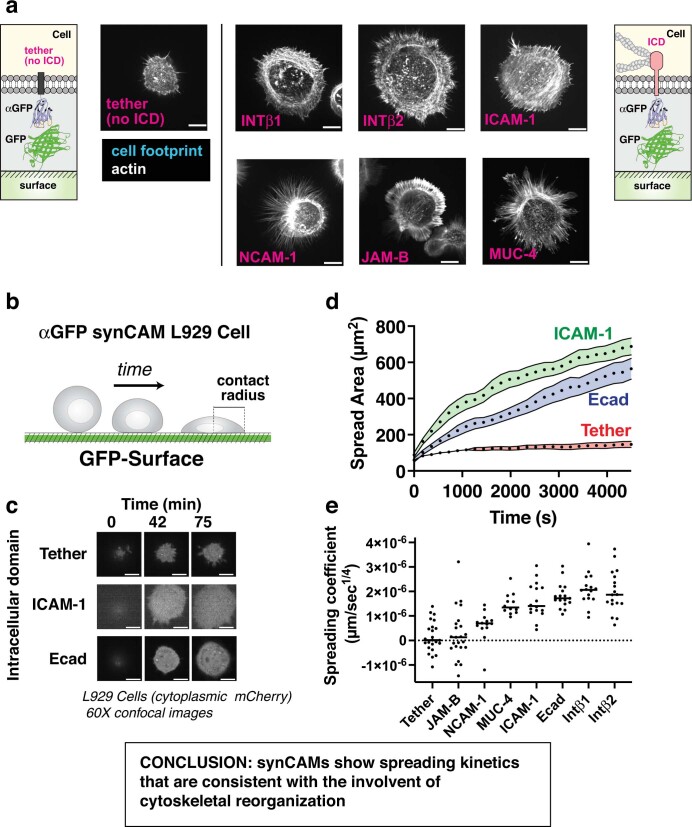
Cell spreading with alternative synCAMs (linked to Fig. 2). (a) Example microscopy images of cell spreading assays from Fig. 2, showing phenotypes for all synCAM species (Scale bar = 10 µm). Representative images are shown of independent replicates from Tether n = 10, ICAM-1 n = 20, JAM-B n = 20, MUC-4 n = 15, NCAM-1 n = 20, Intβ1 n = 20, Intβ2 n = 20. SynCAMs are expressed in L929 fibroblasts and plated on a GFP coated glass surface. Cell footprint detected by membrane dye is indicated in blue outline; actin as stained by Phalloidin and shown in white. (b) Cartoon depicting the cell-spreading assay. L929 cells expressing an αGFP synCAM are plated on a GFP-coated surface and monitored over time. (c) Represented images from cell spreading assay of L929 cells expressing the indicated synCAMs. Individual slices from confocal images are shown. Scale bar = 10 µm. (d) Representative cell spreading contact area progress curves of L929 cells expressing the indicated synCAMs. Error = SEM. (e) Calculated spreading constants for L929 cells expressing the indicated synCAMs (where n is the number of unique cells analysed, Tether n = 24, Ecad n = 17, JAM-B n = 23, ICAM-1 n = 16, Intβ1 n = 16, Intβ2 n = 18, NCAM-1 n = 14, MUC-4 n = 12. Indicated line represents the median value). Source data

To examine synCAM interfaces in more detail, we used a more controlled assay in which an L929 cell expressing an anti-GFP synCAM interacts with a GFP-coated surface (Fig. 2 and Extended Data Fig. 5a). Here, because surface GFP is immobile and cannot rearrange, the interacting synCAM cells spread on the surface. After 75 min, cells were fixed and stained with Phalloidin to observe the actin cytoskeleton. A simple anti-GFP–tether interaction yielded minimal cell spreading on the GFP surface (Fig. 2a). However, synCAMs once again showed two distinct modes of spreading. Cells expressing synCAMs with ICDs from ICAM-1, ITGB1, ITGB2 and ECAD uniformly expanded on the GFP surface, developing a dense band of cortical actin along the cell periphery (Fig. 2b). Kinetic studies show that this larger spreading has a slow phase of tens of minutes to hours, consistent with a requirement for cytoskeletal remodelling (Extended Data Fig. 5b–e). These synCAMs generate uniform ‘expansive’ spreading along the entire periphery of the cell. By contrast, synCAMs with the MUC-4, NCAM-1 and JAM-B ICDs yielded a ‘fried egg’ morphology—a smaller central cell mass was surrounded by thin membrane protrusions at the periphery (Fig. 2c). In these cases, lamellipodial and/or filopodial actin structures mediated radially ‘protrusive’ spreading. Overall, these surface-spreading studies are consistent with our previous cell–cell interface studies, as the expansive spreading synCAMs also lead to larger cell–cell interfaces and greater contact angles, whereas the protrusive spreading synCAMs formed small but highly enriched interfaces.

**Extended Data Fig. 6:**
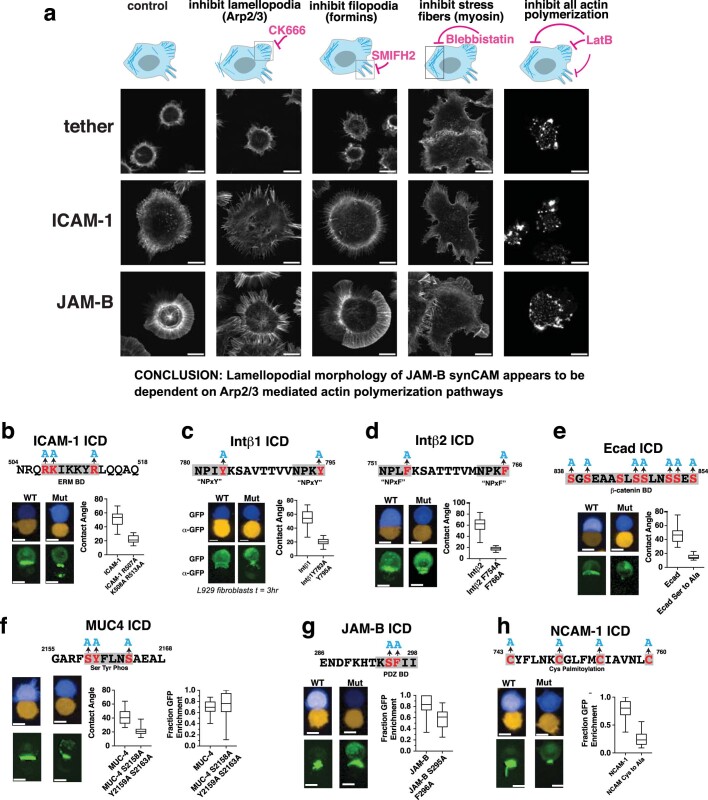
How synCAM morphology is perturbed by small molecule inhibitors of specific actin regulators and loss of function mutations (linked to Fig. 2). (a) Example microscopy images of L929 fibroblasts expressing αGFP JAM-B, ICAM-1, or Tether spreading on a GFP coated surface and stained with Phalloidin (scale bar = 10 µm). Spreading is shown in the presence of the indicated inhibitor of actin regulation. A minimum of 10 regions of interest were imaged on two separate days. (b) Maximum projection confocal images (scale bar = 10 µm), and calculated contact angles of synCAM interfaces containing the ICAM-1 ICD with mutations in the ERM binding domains (BD) (box = 25th to 75th percentile, whiskers = min to max, centre = median, n = 20 pairs)^60^. (c) Maximum projection confocal images (scale bar = 10 µm), and calculated contact angles of synCAM interfaces containing the Intβ1 ICD with mutations in the two “NPxY” talin binding domain motifs (box = 25th to 75th percentile, whiskers = min to max, centre = median, n = 20 pairs)^61^. (d) Maximum projection confocal images (scale bar = 10 µm), and calculated contact angles of synCAM interfaces containing the Intβ2 ICD with mutations in the two “NPxF” talin binding domain motifs (box = 25th to 75th percentile, whiskers = min to max, centre = median, n = 20 pairs)^62^. (e) Maximum projection confocal images (scale bar = 10 µm), and calculated contact angles of synCAM interfaces containing the Ecad ICD with mutations in the β -catenin binding domain (box = 25th to 75th percentile, whiskers = min to max, centre = median, n = 20 pairs)^63^. (f) Maximum projection confocal images (scale bar = 10 µm), and calculated contact angles and GFP enrichment of synCAM interfaces containing the MUC-4 ICD with mutations in Ser and Tyr phosphorylation sites (box = 25th to 75th percentile, whiskers = min to max, centre = median, n = 20 pairs). (g) Maximum projection confocal images (scale bar = 10 µm), and calculated contact angles and GFP enrichment of synCAM interfaces containing the JAM ICD with mutations in the PDZ binding domain (box = 25th to 75th percentile, whiskers = min to max, centre = median, n = 20 pairs). (h) Maximum projection confocal images (scale bar = 10 µm), and calculated contact angles and GFP enrichment of synCAM interfaces containing the NCAM-1 ICD with mutations in the Cys palmitoylation site (box = 25th to 75th percentile, whiskers = min to max, centre = median, n = 20 pairs)^64^. Source data

We investigated how synCAM-driven cell spreading was altered by a series of small-molecule inhibitors of distinct actin regulators (Extended Data Fig. 6a). All synCAM-expressing cells displayed minimal spreading in the presence of latrunculin B, which disrupts actin filament formation, confirming the importance of cytoskeletal activity in all modes of cell spreading. By contrast, inhibiting contractility with blebbistatin (but still allowing actin polymerization) enabled synCAM cells to spread, but without controlled assembly of actin into distinct structures unique to the different synCAMs. This result emphasizes the competition between spreading and cortical contractility as a cell extends a new interface^24,26^. For protrusive spreading synCAMs (for example, JAM-B ICD), the lamellipodial sheets normally seen at the periphery of the cell are disrupted by CK666, indicating a role of its target, the ARP2/3 complex, in the formation of these thin protrusive structures.

The distinct interface morphologies observed here can be explained by postulated mechanisms of the CAM ICDs (Fig. 2e). Although they individually differ in detail, the expansive ICDs (ECAD, ICAM-1, integrins) recruit adapter molecules such as β-catenin, talin, vinculin and ERM proteins, which are thought to engage the cortical actin cytoskeleton and therefore drive expansion of the entire cell front^12,13,27^. By contrast, the protrusive ICDs (NCAM-1, JAM-B, DLL1) interact with PDZ scaffold proteins or lipid rafts, generally forming organized complexes that involve clustering or phase condensation^28–31^. The resulting spatially focused assemblies may then drive protrusive cytoskeletal responses such as the formation of filopodia and lamellipodia by recruiting and activating proteins such as N-WASP and ARP2/3. The importance of these ICD interaction domains in interface formation was confirmed by mutational analysis of key signalling motifs (Extended Data Fig. 6b–h).

## Asymmetric interfaces

**Fig. 3:**
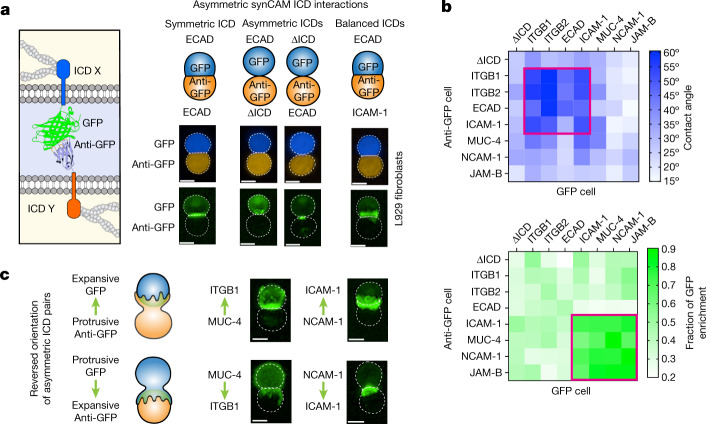
The balance of ICD properties determines asymmetric synCAM interface morphology. **a**, Maximum projection of ×20 confocal microscopy images of pairwise synCAM interfaces (*t* = 3 h), showing symmetric ECAD ICDs (left), asymmetric ECAD and tether (∆ICD) interfaces (middle), and balanced asymmetric ECAD and ICAM-1 interfaces (right). Scale bars, 10 µm. The mCherry and BFP channels (top) and the GFP channels (bottom) of representative images from ten pairs over three independent replicates are shown. **b**, Quantification of the contact angle (top) and GFP enrichment (bottom) for pairwise asymmetric synCAM interfaces. *n* = 10. The combination of interfaces that exhibit the greatest contact angle or enrichment are outlined in red. **c**, Example ×20 confocal microscopy images of pairwise unbalanced asymmetric interfaces in which a protrusive synCAM binds to an expansive synCAM. *t* = 3 h. Scale bars, 10 µm. Representative images from ten pairs over three independent replicates are shown. Top, binding between a protrusive anti-GFP synCAM and an expansive GFP synCAM. Bottom, binding between a protrusive GFP synCAM and an expansive anti-GFP synCAM. Source data

**Extended Data Fig. 7:**
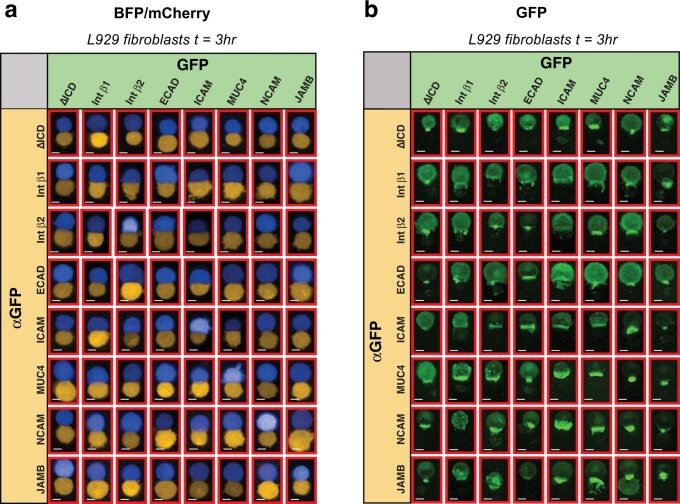
Asymmetric cell–cell interfaces: mismatched ICDs (*linked to main* Fig. 3). (**a**) Maximum projection of 20x confocal microscopy images of pairwise synCAM interfaces (scale bar = 10 µm, t = 3 h): GFP-expressing cell (blue) is bound to an αGFP expressing cell (orange) containing the indicated CAM ICD. Representative images are shown of 10 independent cell pairs. (**b**) GFP channel of cell pairs shown in a.

Many endogenous CAMs bind homophilically (for example, ECAD and JAM-B), yielding an interface with symmetric ICDs. However, many other endogenous CAMs participate in heterophilic interactions (for example, ITGB1, ITGB2 and ICAM-1), leading to cell–cell interfaces with different opposing ICDs. We therefore used the synCAM platform to investigate how symmetric versus asymmetric ICDs affect cell–cell interface morphology. We examined all of the possible pairs of different GFP–anti-GFP synCAMs in L929 fibroblasts (Fig. 3 and Extended Data Fig. 7).

Asymmetric interfaces with a fully deleted ICD (tether) on one side of the interface exhibit significantly disrupted interfaces—they show minimal cell–cell interface expansion and contact angle increase (Fig. 3a,b). However, a large asymmetric interface can be formed if it pairs two expansive synCAMs (for example, ECAD–ICAM-1 or ECAD–ITGB2) (Fig. 3a,b). These findings suggest that large, expanded interfaces can form with asymmetric synCAMs if the opposing ICDs yield a balanced interaction. Analogously, asymmetric interfaces pairing two ICDs that both mediate GFP enrichment (for example, NCAM-1–MUC-4, NCAM-1–JAM-B) generate an interface enrichment phenotype that is similar to that of symmetric interfaces (Fig. 3a,b). Thus, to form a productive interface, the exact sequence of an opposing ICD is less critical than the presence of ICDs with matched strength and morphology.

Notably, when we created heterotypic interfaces in which a cell with a protrusive synCAM binds to a cell with an expansive synCAM, the cells interacted with a consistent morphology—they formed an asymmetric interface in which the protrusive synCAM cell wraps around the expansive synCAM cell (Fig. 3c). These results show the diversity of interfaces that can be constructed with synCAM combinations.

## Programming de novo cell assembly

**Fig. 4:**
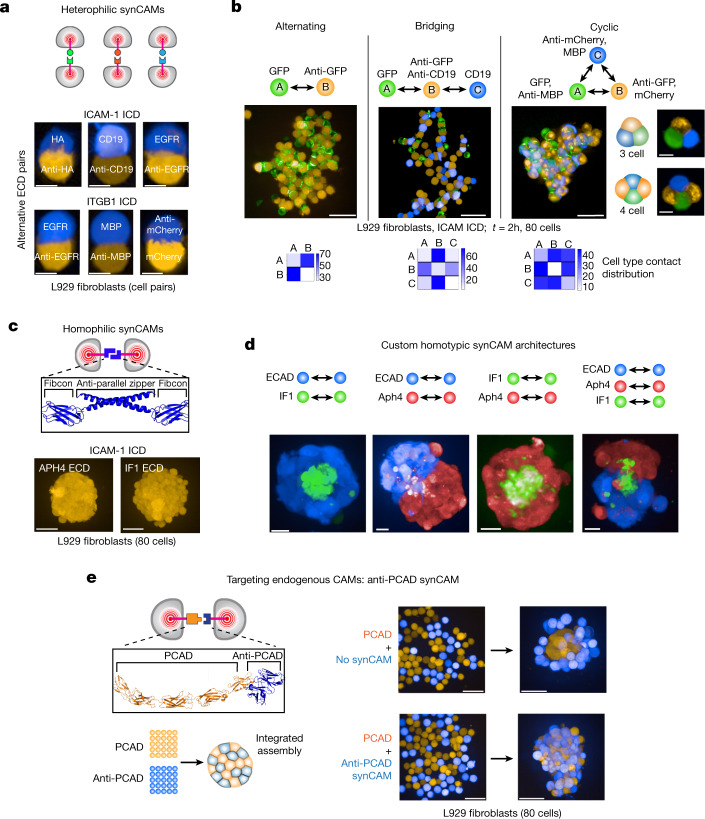
Programming custom multicellular assemblies with homotypic and heterotypic synCAMs. **a**, Heterophilic synCAMs with orthogonal extracellular recognition domains. Maximum projection of ×20 confocal microscopy cell–cell interface images are shown of L929 cells expressing synCAMs with the indicated antibody–antigen pair ECDs and either ICAM-1 (top) or ITGB1 (bottom) TM/ICD. Scale bars, 10 µm. *t* = 3 h. Representative images are shown of four independent replicates. Experimental testing of orthogonal sorting is shown in Extended Data Fig. 8. See Supplementary Video 1 for a time-lapse analysis of the orthogonal assembly formation. **b**, Engineering of custom heterotypic assemblies. Maximum projection of ×20 confocal microscopy images of L929 cells expressing synCAMs with the indicated ECD recognition partners. *t* = 2 h. Assemblies form alternating (left), bridging (middle) and cyclic (right) patterning. Example images of isolated cyclic interactions are shown. *t* = 2 h. A time-lapse analysis is shown in Supplementary Video 2. Probability boxes of cell contact distribution are shown below. *n* = 5. Scale bars, 50 µm (left 3 images) and 10 µm (insets). **c**, synCAM design with a homophilic-binding leucine zipper ECD (top). Bottom, maximum projection of ×20 confocal microscopy images of L929 cells expressing homophilic-binding synCAMs with the Aph4 or IF1 leucine-zipper ECD and ICAM-1 TM/ICD (ULA round-bottom well, 80 cells total). Scale bars, 50 µm. *t* = 24 h. Representative images are shown of three independent replicates. **d**, ×20 confocal microscopy images of differential sorting between L929 cells expressing WT ECAD or the indicated homophilic-binding synCAMs. Scale bars, 20 µm. *t* = 48 h. Representative images are shown with additional independent replicates in Extended Data Fig. 9. *n* = 15 (ECAD–IF1), *n* = 15 (ECAD–Aph4), *n* = 14 (IF1–Aph4) and *n* = 18 (ECAD–IF1–Aph4). **e**, Schematic of the receptor design and differential sorting assay of L929 cells expressing WT PCAD (orange) and an anti-PCAD synCAM (anti-PCAD, blue) (left). The anti-PCAD synCAM contains an ICAM-1 TM/ICD. Right, maximum projection images of the sorting assay in which L929 cells expressing WT PCAD (orange) were mixed with parental (top) or synCAM (bottom) L929 cells (blue). Scale bars, 50 µm. *t* = 0 h and 24 h. Representative images are shown of four independent replicates with additional replicates shown in Extended Data Fig. 10. Source data

**Extended Data Fig. 8:**
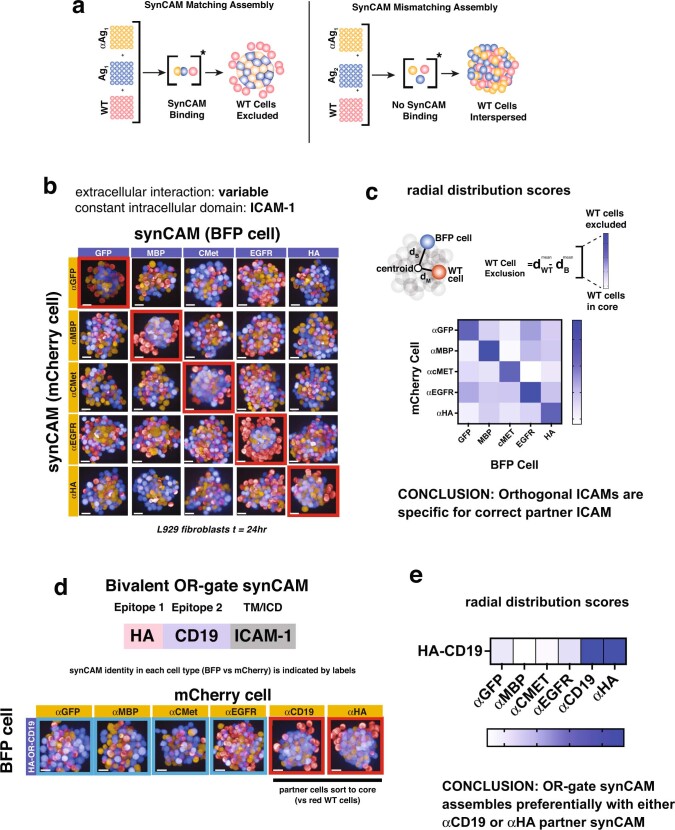
Testing orthogonality of synCAM ECD pairs by sorting assays (*linked to main* Fig. 4a). (**a**) Cartoon depicting differential sorting assay used to determine orthogonality of synCAM ECD pairs. SynCAM pairs are mixed with parental L929 cells and imaged after 24 h. Sorting of parental cells should only occur if the cognate synCAM ECDs are correctly matched and able to bind. (**b**) Representative maximum projection images of differential sorting assay for a subset of the synCAMs with orthogonal ECDs (scale bar = 20 µm). Parental L929 cell sorting was only observed in the case of matching ECDs. (**c**) Quantification of sorting from **b** (n = 6). The difference of average distance from the centre of the sphere between parental L929 cells and BFP+ cells were calculated and are represented as a heat map. Exclusion of parental cells is observed in the case of matching synCAM pairs. (**d**) Representative maximum projection images synCAM design containing multiple epitopes within a single ECD (scale bar = 20 µm). The HA-CD19 ECD exhibits differential sorting for either αCD19 or αHA synCAMs only. Thus, we can generate OR-gate synCAMs capable of pairing with multiple adhesion partners. (**e**) Quantification of sorting from **d** (n = 6). The difference of average distance from the centre of the sphere between parental L929 cells and BFP+ cells were calculated and are represented as a heat map. Exclusion of parental cells is observed in the case of matching synCAM pairs. Source data

Programming the formation of new multicellular tissues de novo requires dictating specific cellular connectivity within a multicellular system^5,32,33^. Previous efforts to orthogonally control multicellular assembly, both in bacteria and mammalian systems, have generally used surface-tethering approaches^5,32–35^. Notably, recent research has enabled custom patterning of engineered bacteria through the surface expression of orthogonal nanobody–antigen pairs^33^. Given the ability of synCAMs to direct cellular morphology and cytoskeletal structure, we tested whether synCAMs could be engineered with a wide range of orthogonal ECDs to also rationally program specific spatial connectivity. We found that functional synCAMs could be built with multiple distinct antibody–antigen binding pairs, including HA-tag–anti-HA single-chain variable fragment (scFv); maltose-binding protein (MBP)–anti-MBP nanobody; B cell surface antigen CD19–anti-CD19 scFv; tyrosine-protein kinase MET–anti-MET nanobody; mCherry–anti-mCherry nanobody; and epidermal growth factor receptor (EGFR)–anti-EGFR nanobody (Fig. 4a and Supplementary Video 1). The orthogonality of distinct ECD synCAMs was confirmed using co-sorting assays, and we quantified their efficiency in excluding WT L929 cells from the multicellular assembly (Extended Data Fig. 8).

We tested whether this set of orthogonal heterotypic synCAMs could program highly specific cell bonding patterns. (Fig. 4b and Supplementary Video 2). We constructed assemblies with the following patterns: (1) two cell (A↔B) alternating heterophilic interactions (expression of a heterophilic GFP–anti-GFP synCAM pair in cells A and B); (2) three cell (A↔B↔C) bridging interactions (expression of orthogonal synCAMs in cells A and C, and both complementary synCAMs in the bridging cell B); (3) three cell (A↔B↔C↔A) cyclic interactions (expression of two orthogonal synCAMs in each of cells A, B and C). The resulting assemblies organize as dictated by the synCAM-defined cell–cell connectivities. Nearest neighbour distribution analysis (using Harmony image analysis software) showed that synCAM-specified interactions dominate assembly (Fig. 4b). In magnified images with low numbers of cells, the cyclic interaction set can lead to the predicted minimal 3 and 4 multicell assemblies (Fig. 4b). Thus, synCAM combinations can specify the precise bonding connectivities between cells.

We next engineered homotypic synCAMs from self-dimerizing coiled-coil ECD interactions. We used the Aph4 (a computationally designed leucine zipper^36^) and the IF1 (bovine ATPase inhibitor IF1) leucine zippers, as we anticipated that their antiparallel binding topologies might sterically favour intercellular *trans*-cell interactions over intracellular *cis* binding^36,37^. We also appended an intervening fibcon domain (a consensus FN3 domain from fibronectin) adjacent to the coiled-coil domains to provide additional separation from the juxtamembrane region, which could further favour *trans* cell interactions^38^ (Fig. 4c).

**Extended Data Fig. 9:**
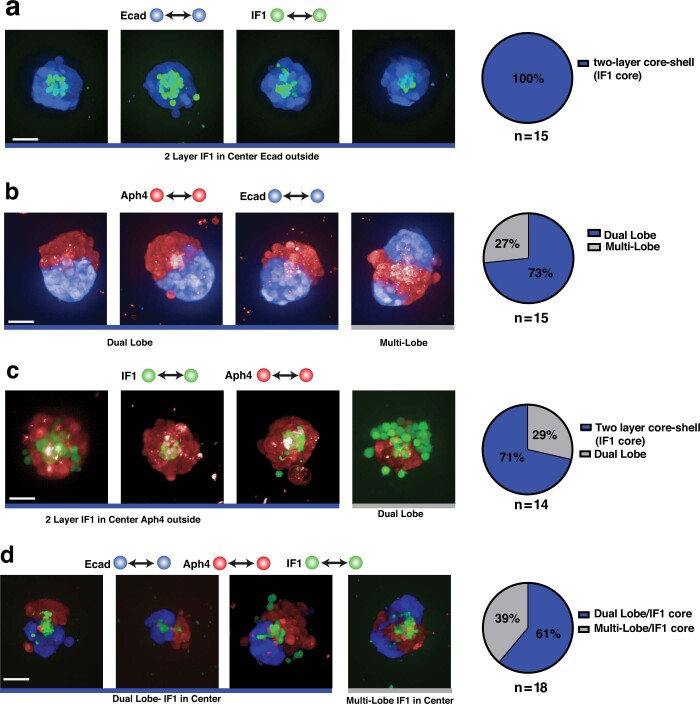
Replicates and distribution of assemblies formed from custom homotypic synCAMs (linked to Fig. 4d). Maximum projection of 20X confocal microscopy images of differential sorting between L929 cells expressing WT Ecad or the indicated homophilic-binding synCAMs (scale bar = 50 µm, t = 48 h). Representative images, assembly classifications and distributions are shown for Ecad-IF1 (a), Ecad-Aph4 (b), IF1-Aph4 (c) and Ecad-IF1-Aph4 (d).

We tested whether cells expressing orthogonal homophilic synCAMs could predictably generate structures with segregated compartments. Cells expressing three different orthogonal homotypic CAMs (WT ECAD, Aph4–ICAM-1 or IF1–ICAM-1) were mixed in combinations (Fig. 4d) and classified on the basis of the resulting assembly structures. The individual cell populations show clear sorting through their homophilic synCAMs, but most striking is the highly modular sorting behaviours that result. When cell types were mixed in a pairwise manner, we observed that the IF1 cells sort to the centre versus ECAD or Aph4. The ECAD and Aph4 cells sort into a two-lobed barbell structure. These relationships are maintained when all three cell types are mixed, yielding a structure with an ECAD–Aph4 barbell cell assembly with IF1 cells at the core (Extended Data Fig. 9 for assembly statistics). These results show how a toolkit of orthogonal synCAMs can build multicompartment self-organizing structures with modularity and predictability.

## Intercalation into native assemblies

**Extended Data Fig. 10:**
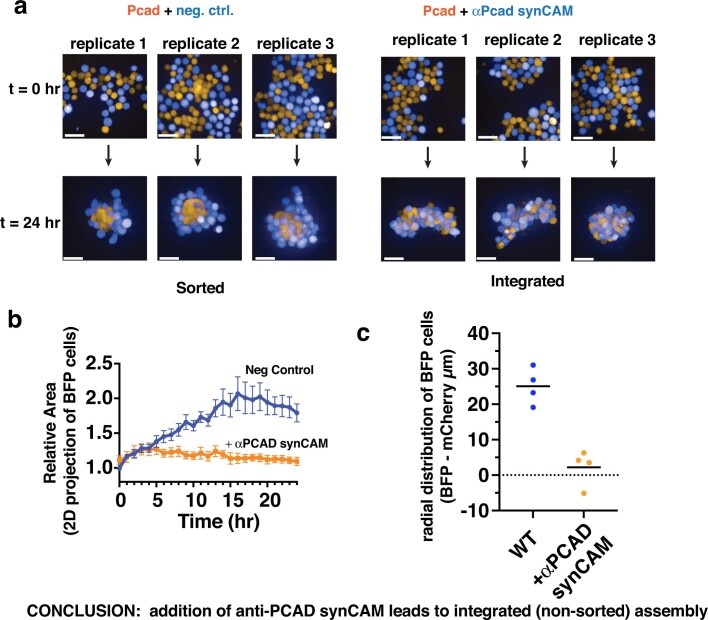
Targeting WT Pcad with synCAM (*linked to main* Fig. 4e). (a) Maximum projection images of the sorting assay in which L929 cells expressing WT Pcad (orange) are mixed with parental (left) or synCAM (right) L929 cells (blue, t = 0, 24 h, scale bar = 50 µm). (b) Quantification of the relative area between the BFP negative control or αPcad synCAM and mCherry (Pcad) L929 cells over the course of the 24-h assembly (n = 4 biologically independent samples, error = SEM). A greater difference in area is consistent with a more compact Pcad sphere and exclusion of BFP+ cells. (c) Quantification of relative distance per cell (BFP-mCherry) from the centre of the sphere following assembly (t = 24 h, n = 4 biologically independent samples, line= mean). WT BFP cells exhibit a greater difference in distance, which is consistent with their exclusion from the Pcad sphere, while αPCAD synCAMs intercalate. Source data

We tested whether synCAMs could directly interface with a tissue held together by native adhesion molecules such as P-cadherin (PCAD). Thus, we engineered a synCAM with an anti-PCAD scFv fused to the ICAM-1 ICD (Fig. 4e, Extended Data Fig. 10 and Supplementary Video 3). These synthetic PCAD-targeting cells could effectively intercalate into a cell spheroid held together by PCAD. By contrast, cells lacking the synCAM were excluded and sorted to the exterior of the structure. Thus, synCAMs can integrate cells into assemblies formed by native adhesion molecules.

## Use in primary and iPS cell-derived cells

**Extended Data Fig. 11:**
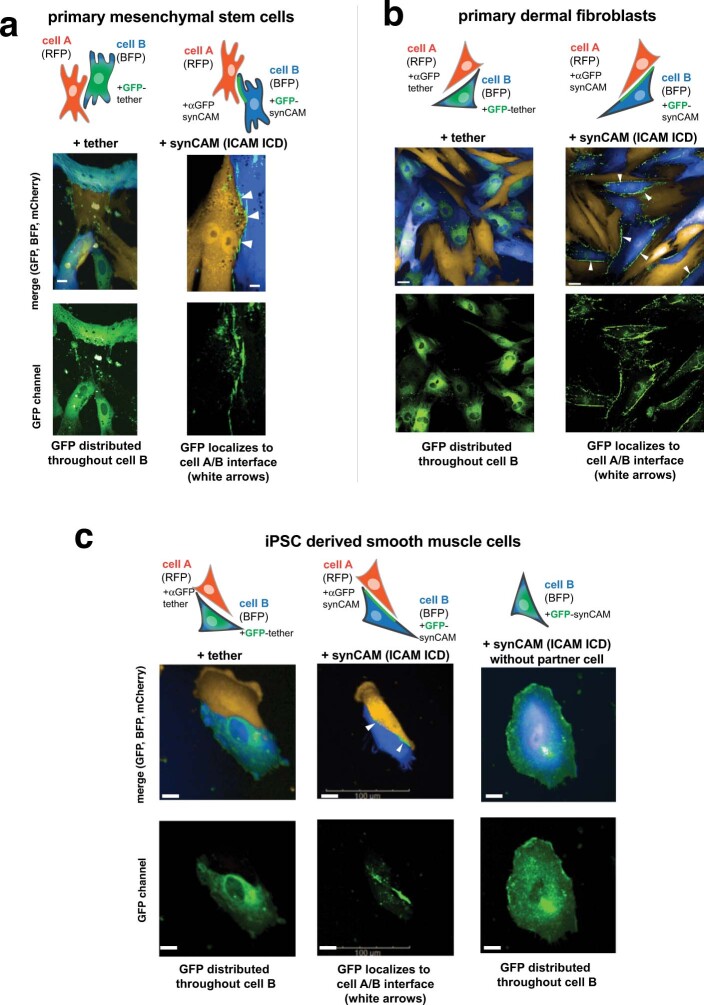
SynCAMs function in primary cells: mesenchymal stem cells (MSCs), dermal fibroblasts, and iPSC derived smooth muscle cells (*linked to main* Fig. 4). Maximum projection of 20x confocal microscopy images of αGFP and GFP synCAMs (with ICAM-1 ICD) or corresponding tether (no ICD) expressed in MSCs (a, scale bar = 10 µm) primary dermal fibroblasts (b, scale bar = 20 µm) or iPSC derived SMCs (c, scale bar = 20 µm). αGFP cells were also labelled with mCherry; GFP cells were also labelled with BFP. Representative images are shown of three independent replicates. In both cell types, the GFP-tether is diffusely spread throughout the cell. In contrast, the GFP-synCAM is strongly enriched atheterotypic cell–cell interfaces (white arrows). When cells expressing GFP-synCAMs are plated without their partner cells, the GFP is diffusely distributed throughout the cell.

We tested whether synthetic adhesion molecules could function in primary cells and induced pluripotent stem (iPS) cell-derived cells. GFP/anti-GFP–ICAM-1 synCAMs and GFP/anti-GFP–tether molecules were expressed in several primary or iPS cell-derived cells (Extended Data Fig. 11). When ICAM-1-based synCAMs are expressed in primary human dermal fibroblasts, human mesenchymal stromal cells and iPS cell-derived smooth muscle cells, we observed strong localization of the GFP-tagged synCAMs to the interface formed with partner cells expressing a functional cognate anti-GFP synCAM. This synCAM relocalization to the heterotypic cell–cell interface was not observed either in unbound cells (GFP synCAM remains distributed throughout cell, not just at the interface) or when co-cultured with partner cells contained only an anti-GFP–tether (no ICD). These results demonstrate that synCAMs functionally engage each other in these different cell types in a manner that is dependent on cognate ECDs and the presence of functionally matched ICDs.

## Remodelling tissue organization

**Fig. 5:**
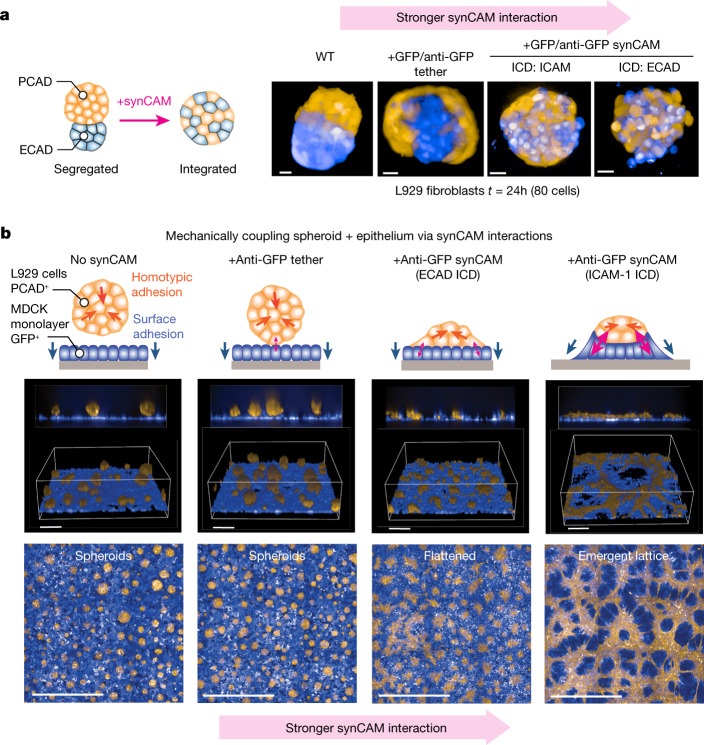
Using synCAMs to reshape tissue organization. **a**, Schematic of the experiment using synCAMs to force the integration of differentially sorting L929 populations. We start with L929 populations expressing WT ECAD (blue) or WT PCAD (orange), which leads to segregation into a binodal structure. The image shows how sorting is altered by the expression of integrating heterophilic synCAM with interactions of different strengths (versus the tether receptor). Maximum projections of ×20 confocal microscopy images are shown. Scale bars, 20 µm. *t* = 24 h. See Supplementary Video 3 for a time-lapse analysis. A similar demonstration of synCAM integration of PCAD/NCAD-segregated cell populations is shown in Extended Data Fig. 7. **b**, L929 cells expressing WT PCAD (orange) mixed with an MDCK monolayer (blue) form spheroids that passively sit above the MCDK epithelial layer. Adding GFP–anti-GFP synCAMs with interactions of increasing strength (versus the tether receptor) leads to increasing mechanical coupling between the epithelial and spheroid tissues. When strong enough, the two cell types form a complex lattice-like network (ICAM synCAM). The images show assembly at *t* = 24 h. Both 3D magnified (top) and maximum projection zoomed-out (bottom) views are shown. Scale bars, 100 µm (top) and 1 mm (bottom). See Supplementary Video 4 for a time-lapse analysis of coupled tissue evolution.

**Extended Data Fig. 12:**
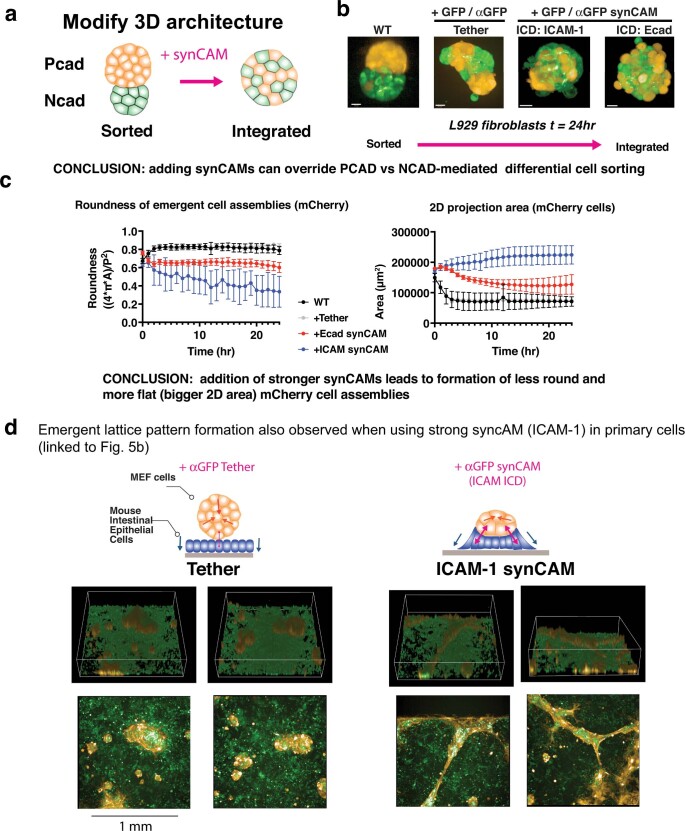
Control of Multicellular organization by synCAMs (*linked to main* Fig. 5). (a) cartoon depicting modulation of WT Ncad (green) and WT Pcad (orange) sorting through introduction of synCAMs. (b) Maximum projections of 20X confocal microscopy images of WT Pcad and WT Ncad L929 cells with expression of the indicated heterophilic synCAMs (scale bar = 20 µm, t = 24 h). The GFP-synCAM is expressed in the Ncad-expressing L929 cell and αGFP synCAM in the Pcad-expressing L929 cell. Representative images are shown of three independent replicates. This data shows that synCAMs can drive integration between differentially sorting Pcad and Ncad cells, just as they can between Pcad and Ecad cells (Fig. 5a). (c) Quantification of roundness (left) and total surface area (right) of L929 cells from maximum projections of 20x confocal images in Fig. 5b (data are presented as mean values of n = 18 unique fields analysed across two independent wells, error = SD). (d) 3D (top) and maximum projection (bottom) views of multicellular assemblies between a mouse intestinal epithelial monolayer (green) and mouse embryonic fibroblast cells (MEFs) (orange) with either a GFP-αGFP tether (left) or synthetic ICAM-1 (right) heterophilic adhesion interaction. Representative images are shown of two independent replicates. Source data

We examined whether synthetic adhesion could remodel and reconfigure multicellular tissues organized by native CAMs. For example, L929 cells expressing WT ECAD and WT PCAD differentially sort from each other into a bilobed assembly^6^. We examined whether introducing a GFP–anti-GFP synCAM interaction could force these two segregating populations to integrate (Fig. 5a). Expression of a heterotypic tether molecule converted the bilobed assembly into a two layered (core shell) structure, which maintains segregation, but slightly increases the number of heterophilic contacts relative to the bilobed assembly. By contrast, expression of the stronger synCAMs (ICAM-1 or ECAD ICDs) converted the bilobed structure into an integrated structure, with the two cell types in a single mixed compartment. These synCAMs could also force integration of differentially sorted L929 cell populations expressing WT PCAD or WT NCAD (Extended Data Fig. 12a,b). Thus, synCAMs can be used to systematically remodel multicell assemblies.

To further examine tissue remodelling, we tested whether synCAMs could alter epithelial monolayers—a fundamental building block for diverse tissues and organs. For example, modulation of epithelial structure by interactions with mesenchymal cells is a common theme in development. We used Madin–Darby canine kidney (MDCK) cells as a starting epithelial cell layer. When a population of L929 cells expressing PCAD are added, they form segregated homotypic spheroid clusters that sit above the confluent MDCK epithelial layer. The starting epithelial (MDCK) and spheroid (PCAD-L929) tissues show minimal interactions, functioning as independent assemblies (Fig. 5b).

We examined whether introducing bridging synthetic adhesion interactions (using GFP–anti-GFP ECDs with symmetric ICDs) could force the distinct epithelial and spheroid tissues to interact. When a minimal tether interaction (no ICD) is added, the PCAD-L929 cells sit tightly on the MDCK epithelial layer, but still act independently, maintaining their segregated spheroid structure. However, introducing a stronger ECAD synCAM results in the PCAD-L929 spheroids spreading into flatter, aster-like bumps that more extensively contact the epithelial layer. Finally, adding the even stronger ICAM-1 synCAM bridging interaction causes substantial cooperative rearrangement of both tissues (Fig. 5b, Extended Data Fig. 12c and Supplementary Video 4). In this case, L929 cells organize into a continuous lattice network on top of the MDCK cells. Moreover, the MDCK epithelial layer shows reduced confluence, perhaps because the strong bridging interaction between the L929 and MDCK cells appears to pull up MDCK cells from the surface in the intervening spaces of the lattice. We hypothesize that this cooperative tissue emerges from the opposing forces of the two tissues. The strong homotypic (PCAD) attraction among the L929 cells combined with the strong synthetic bridging interaction (synCAM) between the L929 cells and the MDCK cells results in these two populations adopting a mechanically balanced state. The resulting network is reminiscent of the self-organizing capillary tube network of activated endothelial cells. In brief, this lattice configuration appears to provide a solution that enables the L929 cells to simultaneously maintain a high degree of homotypic interaction, along with a high degree of heterotypic interaction with the MDCK epithelial layer. A similar emergent lattice network structure was observed in an analogous experiment in primary cells (primary mouse intestinal epithelial layer plus mouse embryonic fibroblasts; Extended Data Fig. 12d). In summary, synCAMs can systematically couple otherwise independent cell populations to yield multicell systems of which the cooperative mechanics yield complex tissue structures.

## Discussion

Here we reveal the potential for engineering diverse synthetic adhesion molecules that share the design principles of native adhesion molecules, but that specify new and orthogonal connectivities between cells. Although metazoans deploy a plethora of CAMs to mediate diverse cellular interactions and tissue assembly, many more interfaces probably remain untapped by evolution. The synCAM design strategy used here integrates two mechanisms for controlling synthetic adhesion. First, the extracellular interaction domain specifies cell–cell connectivity (bonding), which can be either homophilic or heterophilic with precisely controlled affinity. Second, the intracellular domain dictates cytoskeletal reorganization and largely determines the interface mechanics and morphology. The orthogonality and tunability of extracellular domain recognition coupled to the modularity of intracellular domain output expands the possible set of interfaces that can be generated. This toolkit can therefore alter both cell–cell connectivity and the resulting interface type. Furthermore, mixing multiple synCAMs and native CAMs to create a system of mechanically coupled cells can generate tissues with complex emergent structures.

The broad spectrum of adhesion ICDs amenable to chimeric engineering demonstrates that intracellular domain function is, to some degree, independent of the endogenous extracellular recognition mechanism. Notably, the simple extracellular interactions used here do not match the higher regulatory sophistication of many natural ECDs, which can also show *cis*-oligomerization, catch bonding and allosteric changes^8,39–42^. Nonetheless, synCAMs are still sufficient to assemble similar cell–cell interfaces. The modularity of CAMs provides insights into how many natural CAMs may have evolved. For example, proteins with cadherin ECDs are found in choanoflagellates (the closest single-cell relatives to metazoans), but they lack the metazoan ICDs^43,44^. These proteins may have been used by choanoflagellates to bind to food or substrates rather than for cell–cell adhesion, and then later co-opted for cell–cell adhesion through recombination with intracellular signalling domains^43^.

This study supports a dominant role of the intracellular domain in dictating the character of CAM-mediated cell–cell interfaces. Tethering interactions between cells that do not engage the cytoskeleton are unable to generate strong, extensive interfaces, no matter what the extracellular binding affinity is. By contrast, synCAMs consisting of ICDs that engage the cytoskeleton facilitate a more complex morphology that depends on the identity of the ICD on each side of the interface. These observations are consistent with previous studies suggesting that cadherin ICDs remodel cortex tension to drive cell interface expansion and resistance to cell separation^23,24,45–48^.

Finally, we show that synCAMs provide a versatile toolkit for programming multicellular structures, either de novo or by intercalating or remodelling tissues formed by native CAMs. The toolkit of synCAMs also enables systematic perturbation of self-organizing systems that could be used to analyse the mechanism of diverse developmental processes. In the future, these types of engineered adhesion molecules could potentially be applied to address therapeutic problems such as to precisely direct tissue repair and regeneration or to control the interactions and trafficking of immune and neural cells^49^﻿.

## Methods

### Materials

Oligonucleotides were purchased from Integrated DNA Technologies. In-Fusion cloning reagent, CloneAmp HiFi PCR Premix, the Lenti-X concentrator kit and Stellar chemically competent cells were purchased Takara Bio. Miniprep kits and spin columns were purchased from Qiagen. FuGENE HD Transfection Reagent was purchased from Promega. DMEM, GlutaMAX, Alexa Fluor 647 Phalloidin (A22287) and Alexa Fluor 555 Phalloidin (A34055) were purchased from Thermo Fisher Scientific. Fetal bovine serum (FBS) was purchased from the University of California, San Francisco (UCSF) Cell Culture Facility. L929 mouse fibroblast cells (ATCC, CCL-1) were purchased from the American Type Culture Collection. MDCK cells were a gift from the Mostov laboratory at UCSF. Primary dermal fibroblast cells (CC-2511), mouse embryonic fibroblasts (M-FB-481) and human bone-marrow-derived mesenchymal stem cells (PT-2501) were purchased from Lonza Bioscience. Nexcelom 3D 384-well ultra-low attachment treated round-bottom multi-well plates were purchased from Nexcelom Bioscience. Cellstar Cell-Repellent Surface 384-Well flat-bottom plates were purchased from Greiner Bio-One. The 384-well optical imaging flat clear-bottom TC-treated plates were purchased from Corning. H9 human pluripotent stem cells (hPSCs) (WA09) were purchased from WiCell. EDTA (46-034-CI) and growth-factor-reduced Matrigel (356231) were purchased from Corning (Corning). Geltrex, hESC-qualified (A1413302), Essential 8 Flex Medium Kit (A2858501), Essential 6 Flex Medium Kit (A1516401) and Advanced DMEM/F12 (12634028) were purchased from Thermo Fisher Scientific. Recombinant human/mouse/rat activin A protein (338-AC-050) was purchased from R&D Systems. FBS for iPS cells (1701) was purchased from ScienCell. CellMask deep red plasma membrane dye was purchased from Invitrogen. Phalloidin-iFluor 405 Reagent (ab176752) was purchased from Abcam.

The following antibodies were purchased and diluted in PBS before use according to the manufacturer’s protocol: DYKDDDDK epitope tag Alexa Fluor 647-conjugated antibodies (1042E, rabbit, R&D Systems, IC8529R, AEOB0118081, 1:100); DYKDDDDK epitope tag Alexa Fluor 488-conjugated antibodies (1042E, rabbit, R&D Systems, IC8529G, AEOA0521031, 1:100); anti-MYC-tag (9B11) mouse monoclonal antibodies (AlexaFluor 647 conjugate) (Cell Signaling Technology, 2233, 25, 1:100); anti-HA-tag (6E2) mouse monoclonal antibodies (AlexaFluor 647 conjugate) (Cell Signaling Technology, 3444, 15, 1:100); anti-human HGFR/c-MET (95106) AlexaFluor 488-conjugated antibodies (R&D Systems, FAB3582G, 1:50); anti-EGFR antibodies (DH8.3) (AlexaFluor 647) (Novusbio, 50599AF647, 1:50); and anti-6×His tag (HIS.H8) antibodies (Abcam, ab18184, 1:100).

### Equipment

Cell sorting and flow cytometry was performed using the FACSAria II Cell Sorter or LSR II Flow Cytometer (Beckton-Dickinson). Confocal microscopy was performed using the Opera Phenix automated spinning-disk confocal microscope with a ×20 water-immersion objective in 384-well plates; the Nikon TiE with CSU-X1 spinning-disk confocal unit with ×60 and ×100 oil-immersion objectives; or the Zeiss LSM 980 with Airyscan 2 with a ×40 water-immersion objective.

### Synthetic adhesion receptor construct design and cloning

All of the constructs were cloned into a pHR vector containing the SFFV promoter, Kozak consensus sequence and a cleavable signal sequence of influenza haemagglutinin (MKTIIALSYIFCLVFA)^50^.

To design the synCAM constructs, transmembrane and intracellular regions from cellular adhesion molecules were identified from topology annotations in UniProt^51^. Codon-optimized genes encoding each CAM ICD and TM region were purchased from Integrated DNA Technologies and inserted into the vector using In-Fusion cloning. Each CAM TM and ICD region was fused to an extracellular binding domain (for example, GFP, anti-GFP) using In-Fusion cloning (Supplementary Table 2). Sequences for all nanobody or scFv ECDs were obtained from previously reported work or from publicly available patents^20,52–56^. For the experiments involving intestinal epithelial cells, an internal ribosome entry site (IRES) and a puromycin-*N*-acetyltransferase gene (Puro) were cloned downstream of the GFP–ICAM-1 and GFP–tether constructs within the pHR vector. Plasmids were sequence-verified by RF Biotech.

### Lentivirus

Lentivirus was generated by cotransfecting vectors encoding packaging proteins (pMD2.G and p8.91) with the pHR plasmid of interest using the Fugene 6 HD transfection reagent (according to the manufacturer’s protocol) in HEK293T cells plated in 6-well plates at approximately 70% confluence. Two days after transfection, viral supernatants were collected, passed through a 0.45 mm filter and used immediately for transduction.

For transduction of primary cells, lentivirus was concentrated 20-fold using the Lenti-X Concentrator kit (Takara) according to the manufacturer’s protocol.

### Cell lines

L929 and MDCK cells were cultured in DMEM containing 10% FBS. To generate stable cell lines, viral supernatant (50–400 µl) was diluted with 1.5 ml of medium and plated directly with cells (1 × 10^5^ L929 or MDCK) in 12-well dishes. Then, 24 h after infection, the viral medium was replaced with normal growth medium and the cells were expanded into a T25 flask. The cells were stained for the appropriate epitope tag using a fluorescently tagged antibody and sorted for expression by fluorescence-activated cell sorting (FACS). Unless otherwise noted, a bulk-sorted population was used for each experiment. To generate the GFP–ICAM-1 and GFP–tether L929 cell lines with tuned expression levels, total virus added to the cells was titrated between 50 and 400 µl, and the cells were sorted for different synCAM expression levels using FACS. For the Aph4 and IF1 synCAMs, single-cell populations were established by sorting individual cells into a 96-well plate.

### Antibody staining and flow cytometry analysis

To confirm the expression level of synCAMs in each cell line, the cells were analysed using FACS. The cells were detached with TrypLE and transferred to a round-bottom 96-well plate. The cells were pelleted by centrifugation (for 4 min at 400*g*), the supernatant was removed and the cells were resuspended in 40 μl PBS containing a fluorescent-dye-conjugated antibody. Cells were stained for 50 min at 4 °C. The cells were then washed twice with PBS and resuspended in PBS with 5% FBS. The cells were then analysed using flow cytometry (BD LSR II, BD FACSDiva). The flow cytometry data were then analysed using FlowJo (TreeStar).

### Contact angle and receptor enrichment measurements for cell–cell pairs

Before carrying out the experiment, all of the cell lines were detached using TrypLE, resuspended in 1 ml DMEM, counted and then diluted to 4 × 10^5^ cells per ml. L929 cells stably expressing cytosolic BFP and a GFP synCAM were mixed 1:1 with L929 cells expressing cytosolic mCherry and an anti-GFP synCAM in a 384-well flat-bottom plate with a cell-repellent surface (3.2 × 10^4^ cells, 80 µl total volume, 37 °C). At *t* = 3 h, the plates were imaged at ×20 magnification using fluorescence confocal microscopy (Phenix). Maximum-projection images were exported from the manufacturer’s software (Harmony). Distinct cell pairs of similar size were identified, and contact angles were measured in ImageJ. The GFP enrichment percentage was determined in ImageJ by measuring the GFP signal localized at the cell–cell interface as a fraction of that present in the entire cell. Data analysis for the measured contact angle and enrichment values was performed in Prism 9 (GraphPad).

### Cell spreading experiments

We characterized the rate, interface size and morphology of spreading synCAM cells on a GFP-coated surface. Purified GFP protein was diluted to a final concentration of 0.5 µM in PBS and enough volume was applied (~100 µl) to coat the bottom surface of an 8-well glass-bottom imaging chamber. This solution was incubated for 10 min on ice. Excess solution was removed and the chamber was rinsed with PBS. Next, the chamber was blocked with a solution of 10 mg ml^−1^ bovine serum albumin (BSA) and 1 mg ml^−1^ beta casein (Sigma-Aldrich) for a minimum of 1 h on ice. The blocking solution was removed and the chamber was washed three times with PBS. When using CellVis (C8-1.5H-N) chambers, an anti-6×-His antibody (ab18184) and 6×-His-tagged GFP (ab134853) were used to obtain full coverage of the surface with GFP. A 100× dilution of antibody in PBS was incubated on the surface of the chamber for 1 h at 4 °C. After being washed three times with PBS, a 10 µg ml^−1^ solution containing His-tagged GFP was incubated on the surface for 1 h at 4 °C. Next, the chamber was blocked with a solution of 10 mg ml^−1^ BSA and 1 mg ml^−1^ beta casein (Sigma-Aldrich) for a minimum of 1 h on ice. The blocking solution was removed and the chamber washed three times with PBS.

To prepare the cells for the spreading assay, L929 cells were detached using Trypsin EDTA and resuspended in cell culture medium. Around 50 µl of resuspended cell solution from a confluent T25 flask was added to 200 µl of cell culture medium and placed into the imaging chamber. The chamber was then transferred to a spinning-disk confocal microscope equipped with an Oko Labs environmental control stage. Cells were imaged with a ×60 oil-immersion objective every 3 min over a period of 2 h. During the first 60–90 min, spreading of distinct cells onto the surface was observed by monitoring cytoplasmic fluorescent proteins expressed in the cytoplasm of the synCAM cells.

Images were analysed by binarizing the intensity to obtain a mask of the cell, which could then be used to calculate the total spread area (*A*) and perimeter (*p*) of the footprint. To characterize the morphology of the interface, the circularity (*c* = *p*^2^/4π*A*) was calculated and compared between different synCAMs. These measurements were also performed using an anti-flag tag fluorescent antibody (labels synCAM constructs) to measure the area and morphology directly at the interface with the coverslip. To compare different spreading kinetics, the change in area over time was fitted with the following form: *A* = *bt*^1/4^ where *b* is the spreading rate coefficient. This model was previously used to compare the kinetics of spreading cells on an adhesive surface^26^. Analysis was implemented in MATLAB (2020a).

### Immunostaining

To visualize the actin cytoskeleton, spreading cells were fixed and stained for immunohistochemistry according to standard procedures. Cells were fixed in 4% PFA in cytoskeleton buffer (10 mM PIPES, 100 mM NaCl, 300 mM sucrose, 1 mM EGTA, 1 mM MgCl_2_) for 20 min on ice. Cells were then washed three times and permeabilized with 0.1% Triton X-100 solution in PBS for 10 min on ice and again washed three times. Cells were then blocked with 10% BSA in PBS (PBS-BSA) for a minimum of 1 h at 4 °C. To visualize the actin cytoskeleton, cells were stained with fluorescently labelled Phalloidin (conjugated with either Alexa 647, 555 or 405 fluorescent dyes). Cells were then imaged using a spinning-disk confocal microscope using a ×100 magnification objective. Cell peripheries were determined by staining with CellMask deep red plasma membrane stain (Invitrogen). For measurements investigating the effects of cytoskeletal inhibitors on cell spreading, cells were introduced into medium containing the inhibitor and allowed to spread on the GFP-coated surface (CK666 (100 µM), latrunculin B (5 µM), SMIFH2 (100 µM), blebbistatin (50 µM) inhibitors were purchased from Abcam). Cells were then fixed and stained according to the above procedure before being imaged using the Zeiss 980 Airyscan microscope and a ×40 water-immersion objective (Zen Blue).

### Differential sorting assay

Before carrying out the experiment, all of the cell lines were detached using TrypLE, resuspended in 1 ml DMEM, counted and then diluted to 1 × 10^3^ cells per ml. L929 cells stably expressing cytosolic BFP and an anti-GFP synCAM of varying affinity were mixed 1:1:1 with L929 cells expressing cytosolic mCherry and an anti-GFP synCAM of varying affinity, and L929 cells expressing GFP–ICAM-1 in distinct wells of a 384-well ULA round-bottom well (80 µl total volume). At *t* = 24 h, the wells were imaged at ×20 magnification using fluorescence confocal microscopy (Phenix).

### Quantification of the sorting assay

To quantify the organization of different synCAM-expressing cells in the multicellular differential sorting assay, we calculated the radial distribution function *g*(*r*) from multichannel 3D confocal stacks. Cells expressing mCherry and BFP were imaged at ×20 magnification with a *z*-step size of 10 µm. Each slice in the image stack was thresholded and binarized for each colour channel, and the centre of mass (COM) of the cluster was found. *g*(*r*) was found by calculating the distance of each pixel from the COM and normalizing to the density of pixels within the cluster. To create a single value capturing the distribution of cells in the cluster, we calculated the COM of the *g*(*r*) distribution and subtracted this value for the mCherry cells from the value for the BFP cells. Large values therefore indicate that mCherry cells are closer to the centre of the cluster and small values indicate that BFP cells are closer to the centre of the cluster. Image analysis was implemented in MATLAB (2020a).

### Characterization of cell lines expressing orthogonal synCAMs

L929 cells stably expressing synCAMs with orthogonal heterophilic pairs and a cytosolic mCherry or BFP were generated. Before carrying out the experiment, cell lines were detached using TrypLE, resuspended in 1 ml DMEM, counted and then diluted to 4 × 10^5^ cells per ml. Each pair was mixed 1:1 in a 384-well flat-bottom plate with a cell-repellent surface (3.2 × 10^4^ cells, 80 µl total volume, 37 °C). At *t* = 3 h, the plates were imaged at ×20 magnification using fluorescence confocal microscopy (Phenix). Maximum-projection images were generated using the manufacturer’s software.

To validate the orthogonality of the heterophilic synCAM pairs, a subset was characterized for the ability to differentially sort from parental L929 cells. The synCAM cell lines were detached using TrypLE, resuspended in 1 ml DMEM, counted and then diluted to 1 × 10^3^ cells per ml. Parental L929 cells were detached using TrypLE, stained with CellTrace Far Red according to the manufacturer’s instructions, and diluted to 1 × 10^3^ cells per ml. Two synCAMs and the WT L929 cells were mixed 1:1:1 (80 µl total) in a ULA round-bottom well and imaged after 24 h at ×20 magnification using fluorescence confocal microscopy (Phenix). Maximum-projection images were then generated using the manufacturer’s software (Harmony). Within the software, individual cells were segmented, and the centre of the assembly was calculated on the basis of the average position of all cells. The distance of the WT (Far Red) L929 cells and synCAM (BFP) cells from previously calculated centre of the assembly was then determined. The difference between the average distance of WT and synCAM cells was then calculated and represented as a heat map, with greater distances corresponding to increased exclusion of WT cells from the assembly.

### Design and characterization of cell lines expressing homotypic synCAMs

Homotypic synCAMs were designed to sterically impair ECD *cis*-interactions of the binding region. Antiparallel leucine zippers, which should favour *trans* over *cis* binding, were fused to a fibcon linker domain, which extends the receptor from the juxtamembrane region^37,38,36^. Efforts to design homotypic synCAMs without the fibcon linker were unsuccessful. These engineered ECDs were fused to an ICAM-1 TM/ICD.

L929 cells stably expressing the homophilic synCAM receptors and cytosolic mCherry were generated. Clonal cell lines were obtained through single-cell sorting. The cell lines were detached using TrypLE, resuspended in 1 ml DMEM, counted and then diluted to 1 × 10^3^ cells per ml. The cells were incubated in a 384-well ULA round-bottom plate (80 cells, 80 µl total volume, 37 °C) for 24 h and then imaged by fluorescence confocal microscopy (Phenix). Maximum-projection images were generated using the manufacturer’s software (Harmony).

### Targeting endogenous PCAD

L929 cells expressing WT PCAD and cytosolic mCherry were previously generated^6^. L929 cells expressing cytosolic BFP with or without stable expression of an anti-PCAD synCAM (ICAM-1 TM/ICD) were mixed 1:1 with L929 cells stably expressing WT PCAD and cytosolic mCherry in a 384-well ULA round-bottom plate (80 cells, 80 µl total volume, 37 °C) for 24 h and imaged using fluorescence confocal microscopy (Phenix). Maximum-projection images were generated using the manufacturer’s software (Harmony). Within the Harmony software, the total area encompassed by both the L929 cells expressing WT PCAD (mCherry) and the WT or anti-PCAD cells (BFP) was calculated for each maximum-projection image at each timepoint from distinct wells. The ratio of the area for BFP to mCherry cells was then calculated and plotted over 24 h, with an increased ratio corresponding to exclusion of BFP cells from the multicellular assembly (Extended Data Fig. 10b). Moreover, for *t* = 24 h, the cells were segmented and the position of the centre of the assembly was calculated as the average position of the mCherry^+^ and BFP^+^ cells. The relative distance of the BFP^+^ and mCherry^+^ cells to the centre of the assembly was then calculated (Extended Data Fig. 10c) with a greater distance corresponding to increased exclusion of BFP^+^ L929 cells.

### Custom multicellular architecture

For the multicellular patterning experiments, L929 cell lines were detached using TrypLE, resuspended in 1 ml DMEM, counted and then diluted to 1 × 10^3^ cells per ml. Before dilution, the Aph4 and IF1 synCAMs were stained with CellTrace Far Red and CFSE, respectively, according to the manufacturer’s protocol.

#### Heterotypic assemblies

To generate the two-cell alternating pattern, L929 cells expressing GFP–ICAM-1 (cell 1) were mixed with L929 cells expressing cytosolic mCherry and LaG16–ICAM-1 (anti-GFP) (cell 2) (1:1 80 µl total). To generate the three-cell bridging pattern, L929 cells expressing GFP–ECAD (cell 1) were mixed with cells expressing cytosolic mCherry, LaG16–ECAD and anti-CD19–ICAM-1 (cell 2), and cells expressing cytosolic BFP and CD19–ICAM-1 (cell 3) (1:2:1 80 µl total). To generate the three-cell cyclic pattern, L929 cells expressing GFP–ECAD and anti-MBP–ICAM-1 (cell 1) were mixed with cells expressing LaG16–ECAD and mCherry–ICAM-1 (cell 2), and cells expressing MBP–ICAM-1, LaM4–ICAM-1 and cytosolic BFP (cell 3) (1:1:1 80 µl total). In all cases, the cells were plated in ULA round-bottom wells and imaged after 2 h using confocal microscopy (Phenix). Maximum-projection images from distinct wells were generated using the manufacturer’s software (Harmony). To calculate the interaction probability tables, the cells were segmented in Harmony for each maximum-projection image. Cell–cell contacts were identified from the positions of the segmented cells, and the probability for each interaction was calculated and represented as a heat map.

To form the isolated three-cell and four-cell cyclic assemblies, L929 cells expressing GFP–ECAD and anti-MBP–ICAM-1 (cell 1); LaG16–ECAD and mCherry–ICAM-1 (cell 2); and MBP–ICAM-1, LaM4–ICAM-1 and cytosolic BFP (cell 3) were diluted to 4 × 10^3^ cells per ml and plated in a flat-bottom well with a cell-repellent surface. Individual pairs were identified, and maximum-projection images were generated and exported.

#### Homotypic assemblies

L929 cells expressing Wt ECAD and cytosolic BFP, Aph4–ICAM-1 or IF1–ICAM-1 were mixed with each other (either individually or all three together) in ULA round-bottom wells (1:1 or 1:1:1, 80 µl total). The cells were imaged after 48 h using confocal microscopy. The maximum-projection images were generated from distinct wells using the manufacturer’s software (Harmony) and were classified on the basis of assembly phenotype.

### Primary cell culture

Adult human dermal fibroblasts (NHDF-Ad) and mouse embryonic fibroblasts (MEFs) were cultured in DMEM containing 10% FBS. Mesenchymal stem cells (MSCs) were cultured in mesenchymal stem cell growth medium (Lonza).

To generate stable cells expressing the synCAM constructs, viral supernatant (15 µl of ×20 concentrated virus) was diluted with 1.5 ml of medium and plated directly with cells grown to 80% confluency (5 × 10^4^ MSCs, MEFs or NHDFs plated in a 12-well dish). Then, 24 h after transduction, the viral medium was replaced with normal growth medium and the cells were expanded into a 6-well dish. MEFs were further sorted for expression of synCAM constructs by FACS.

### iPS cell-derived smooth muscle cells

Under the official approval from the UCSF Human Gamete, Embryo and Stem Cell Research Committee (GESCR) to F.F., we used the WA09 human embryonic stem cell lines purchased from WiCell in this study. These cell lines and their original sample are completely de-identified and no authors had access to the identifiers.

hPSCs (WA09, WiCell) were maintained in E8 medium on Geltrex-coated six-well plates. Two days before initializing smooth muscle differentiation, hPSCs were dissociated with EDTA and replated into a Geltrex-coated six-well plate. Once hPSCs reached confluency, E8 medium was aspirated and replaced with 1 ml per well of Essential 6 with 100 ng ml^−1^ activin A. The next day, the medium was aspirated and replaced with 2 ml per well of E6 medium with 10 ng ml^−1^ BMP4. Two days later, the medium was aspirated and replaced with 2 ml per well of E6 medium with 10 ng ml^−1^BMP4. For days 5–9, cells were maintained with fresh E6 medium + 2% FBS every other day. From day 10 onwards, the medium was replaced three times per week with Advanced DMEM/F12 + 10% FBS.

To generate SMCs with stable expression of synCAMs, the SMCs were grown to 80% confluency in a 96-well plate and transduced with 1 µl of 20× concentrated virus. After 24 h, the medium was removed and replaced with fresh medium.

### Mouse intestinal epithelial cells

Intestinal epithelium was isolated and cultured as previously described^57^. In brief, small intestinal crypts were dissociated from the duodenum of male C57BL/6 mice aged 6–12 weeks. The tissue was placed into ice-cold PBS with 15 mM EDTA for 30 min, then vortexed vigorously in multiple fractions to release crypts. The supernatant containing the crypts was filtered on a 70 μM mesh, and then the crypts were pelleted and resuspended in growth-factor-reduced Matrigel and cultured as 3D enteroids with ENR medium (Advanced DMEM/F12 (Thermo Fisher Scientific, 12634-028) with 1× N2 (Thermo Fisher Scientific, 17502-048), 1× B27 (Thermo Fisher Scientific, 17504-044), 10 mM HEPES (Thermo Fisher Scientific, 15630080), 1× GlutaMAX (Thermo Fisher Scientific, 35050-061), 1 mM *N*-acetylcysteine (Sigma-Aldrich A9165), 100 U ml^−1^ penicillin and 100 mg ml^−1^ streptomycin (Corning, 30-002), supplemented with 50 ng ml^−1^ EGF (Sigma Aldrich, E9644.2MG), 100 ng ml^−1^ Noggin (R&D 6057-NG/CF), and 5% R-spondin-conditioned medium). The medium was changed every 3 days and organoids were mechanically dissociated and passaged weekly.

For these experiments, mice were maintained in the University of California San Francisco (UCSF) specific pathogen-free animal facility. All maintenance and experiments were performed in accordance with the guidelines established by the Institutional Animal Care and Use Committee and Laboratory Animal Resource Center. All experimental procedures were approved by the Laboratory Animal Resource Center at UCSF. Mice were housed in the UCSF LARC Animal Care Facilities at UCSF Parnassus. They were housed in an individual specific pathogen-free suite. They were housed with up to five mice per cage in ventilator cages, with ad libitum food and water under a 12 h–12 h light–dark cycle and controlled temperature and humidity conditions (20–26 °C and 30–70%).

For expression of synCAM constructs, organoids were transduced with lentivirus as previously described^58^. First, 3D enteroids were dissociated into single cells using TrypLe, which were then grown in growth-factor-reduced Matrigel and transduction medium (NR medium supplemented with 50% Wnt3a-conditioned medium, 10 μM nicotinamide (Sigma-Aldrich, N3376-100G), 5 μM CHIR (Sigma-Aldrich, SML1046-5MG) and 10 μM Y-27632 (Sigma-Aldrich, Y0503-1MG)) for 3–5 days to enrich for stem cells. Enteroids were then dissociated, pelleted, resuspended in transduction medium containing 8 μg ml^−1^ polybrene (Sigma-Aldrich, H9268-5G) and concentrated lentivirus, centrifuged at 600*g* for 1 h at 32 °C, then incubated at 37 °C for 6 h. Cells were then pelleted and resuspended in Matrigel and grown in transduction medium for 3 days, then switched to ENR medium. After amplification, antibiotic selection was performed by adding 1 μg ml^−1^ puromycin (Thermo Fisher Scientific, A1113803) to the medium.

### Primary cell–cell adhesion assays

GFP–ICAM-1, GFP–tether, anti-GFP–fibcon–ICAM-1 or anti-GFP–fibcon–tether were transduced in MSCs, NHDFs or SMCs. For these experiments, a fibcon linker domain was included for both the anti-GFP–ICAM-1 and anti-GFP–tether constructs to improve expression in primary cells. All GFP-expressing cells were co-transduced with a plasmid for expression of cytosolic BFP, and all anti-GFP expressing cells were co-transduced with a construct expressing cytosolic mCherry. Then, 24 h after transduction, the medium was removed and replaced with fresh medium. After 4 to 7 days, the MSCs, SMCs or NHDFs were detached with TryplE, resuspended in medium and plated in a 384-well plate. Then, 24 h after plating, the wells were imaged using fluorescence confocal microscopy (Phenix).

### Modifying 3D architecture

L929 cells stably expressing WT PCAD, cytosolic mCherry and LaG16–synCAM (ICAM-1, ECAD or tether control) were mixed 1:1 with L929 cells stably expressing WT ECAD, cytosolic BFP and a GFP–synCAM (ICAM-1, ECAD or tether control) in a ULA round-bottom plate (80 total cells, 80 µl, 24 h, 37 °C). Before mixing, the L929 cell lines were detached using TrypLE, resuspended in 1 ml DMEM, counted and then diluted to 1 × 10^3^ cells per ml. The assemblies were imaged using fluorescence confocal microscopy (Phenix, ×20 magnification), and maximum-projection images from distinct wells were generated using the manufacturer’s software and are shown.

To modify the assembly between L929 cells expressing WT NCAD and L929 cells expressing WT PCAD, the experiment was performed exactly as described above with L929 cells expressing WT NCAD and cytosolic GFP in place of the WT ECAD cells.

### Modifying 2D layering

An adherent layer of MDCK cells expressing cytosolic BFP and GFP–Tether, GFP–ICAM-1 or GFP–ECAD was formed within wells of a 384-well plate (16,000 cells plated per well). After 48 h, L929 cells expressing WT PCAD, cytosolic mCherry, and LaG16–ICAM-1, LaG16–tether, LaG16–ECAD or no additional receptor were added (24,000 cells per well). The interaction between the two layers was imaged using fluorescence confocal microscopy (Phenix) for 24 h. The zoomed-out images of the assemblies were formed by stitching together nine adjacent fields of view after exporting the images from the manufacturer’s software. Both the roundness and surface area of the mCherry^+^ assembly was quantified for each field of the experiment within the manufacturer’s software (Harmony).

### Modifying 2D layering on intestinal epithelial organoids

Monolayer enteroid cultures were established as previously described^59^. 3D Enteroids were dissociated into single cells using TrypLE, washed in PBS and stained using CellTrace. A total of 150,000 cells expressing either GFP–ICAM-1 or GFP–Tether were plated onto a 384-well pate precoated with 5% growth-factor-reduced Matrigel in 40 μl ENR medium supplemented with 3 μM CHIR and 10 μM Y-27632. After 4 h, an additional 60 μl of ENR medium was added to each well. Then, 24 h after plating, the enteroid monolayers, mouse embryonic fibroblast cells (MEFs) expressing anti-GFP–fibcon–tether or anti-GFP–fibcon–ICAM-1 and cytosolic mCherry were added (16,000 cells). After 24 h, the wells were imaged using fluorescence confocal microscopy (Phenix). Maximum-projection images and 3D images were exported from the manufacturer’s software (Harmony).

### Reporting summary

Further information on research design is available in the Nature Portfolio Reporting Summary linked to this article.

## Online content

Any methods, additional references, Nature Portfolio reporting summaries, source data, extended data, supplementary information, acknowledgements, peer review information; details of author contributions and competing interests; and statements of data and code availability are available at 10.1038/s41586-022-05622-z.

## Supplementary information

Reporting Summary

Supplementary Table 1 SynCAM cell–cell interfaces. Contact angle and GFP enrichment measurements are provided. Error = 95% CI.

Supplementary Table 2 Sequences of protein constructs used in this study. The epitope tag is highlighted in red, the ECD-binding region is shown in blue, and the TM region and ICD in bold.

Supplementary Table 3 SLiM domains from CAM ICDs used in synCAMs. SLiMs were identified from ELM database and literature sources.

Supplementary Video 1 Self-assembly of cells with orthogonal synCAM pairs. Alternative ECD with ICAM-1 ICD are shown (linked to Fig. 4a).

Supplementary Video 2 Three-cell synCAM interaction network. Three-cell synCAM interaction networks drive cell assembly (linked to Fig. 4b).

Supplementary Video 3 SynCAM intercalation into native assemblies. Anti-PCAD synCAM (ICAM-1 ICD) drives cell intercalation into PCAD cluster (linked to Fig. 4e).

Supplementary Video 4 Remodelling tissue organization. SynCAMs can drive coupling and complex remodelling of epithelial and spheroid tissues (linked to Fig. 5b).

## Data Availability

Experimental data supporting the conclusions of this study are available within the Article and the Supplementary Information. All databases used in this study are publicly available. For identifying protein sequences and domain architecture, the Universal Protein Resource (https://www.uniprot.org/) was used. For the identification of linear motifs within CAM ICDs, the Eukaryotic Linear Motif (ELM) resource (http://elm.eu.org/) was used. Additional microscopy replicates are available at Figshare (10.6084/m9.figshare.21647546.v1). Source data are provided with this paper.

## Source data

Source Data Fig. 1
Source Data Fig. 2
Source Data Fig. 3
Source Data Fig. 4
Source Data Extended Data Fig. 2
Source Data Extended Data Fig. 3
Source Data Extended Data Fig. 4
Source Data Extended Data Fig. 5
Source Data Extended Data Fig. 6
Source Data Extended Data Fig. 8
Source Data Extended Data Fig. 10
Source Data Extended Data Fig. 12
