# Supplementary material for: Programming multicellular assembly with synthetic cell adhesion molecules
Source: Nature. 2022 Dec 12;614(7946):144–52. doi: 10.1038/s41586-022-05622-z (PMC9892004; doi:10.1038/s41586-022-05622-z)
Supplement: Supplementary file 1 — Reporting Summary [file 41586_2022_5622_MOESM1_ESM.pdf]

nature portfolio

Corresponding author(s): Wendell Lim

Last updated by author(s): Oct 20, 2022

Reporting Summary

Nature Portfolio wishes to improve the reproducibility of the work that we publish. This form provides structure for consistency and transparency in reporting. For further information on Nature Portfolio policies, see our Editorial Policies and the Editorial Policy Checklist.

Statistics

For all statistical analyses, confirm that the following items are present in the figure legend, table legend, main text, or Methods section.

n/a	Confirmed	
☐	☒	The exact sample size (*n*) for each experimental group/condition, given as a discrete number and unit of measurement
☐	☒	A statement on whether measurements were taken from distinct samples or whether the same sample was measured repeatedly
☒	☐	The statistical test(s) used AND whether they are one- or two-sided<br>*Only common tests should be described solely by name; describe more complex techniques in the Methods section.*
☒	☐	A description of all covariates tested
☒	☐	A description of any assumptions or corrections, such as tests of normality and adjustment for multiple comparisons
☐	☒	A full description of the statistical parameters including central tendency (e.g. means) or other basic estimates (e.g. regression coefficient) AND variation (e.g. standard deviation) or associated estimates of uncertainty (e.g. confidence intervals)
☒	☐	For null hypothesis testing, the test statistic (e.g. *F*, *t*, *r*) with confidence intervals, effect sizes, degrees of freedom and *P* value noted<br>*Give P values as exact values whenever suitable.*
☒	☐	For Bayesian analysis, information on the choice of priors and Markov chain Monte Carlo settings
☒	☐	For hierarchical and complex designs, identification of the appropriate level for tests and full reporting of outcomes
☒	☐	Estimates of effect sizes (e.g. Cohen's *d*, Pearson's *r*), indicating how they were calculated

Our web collection on statistics for biologists contains articles on many of the points above.

Software and code

Policy information about availability of computer code

Data collection	Zen Blue v3.6<br>Micromanager v1.4<br>Harmony 4.9<br>BD FACSDiva (v5B-3R-6V-47G configuration)
Data analysis	FlowJo™ (Mac v10.8.)<br>GraphPad Prism (v9.4.1)<br>Fiji/ImageJ: Version 2.3.0/1.53q , build: d544a3f481<br>MatLab (v2020a)<br>Harmony (v4.9)<br>Zen Blue v3.6

For manuscripts utilizing custom algorithms or software that are central to the research but not yet described in published literature, software must be made available to editors and reviewers. We strongly encourage code deposition in a community repository (e.g. GitHub). See the Nature Portfolio guidelines for submitting code & software for further information.

Data

Policy information about availability of data

All manuscripts must include a data availability statement. This statement should provide the following information, where applicable:

- Accession codes, unique identifiers, or web links for publicly available datasets
- A description of any restrictions on data availability
- For clinical datasets or third party data, please ensure that the statement adheres to our policy

Experimental data supporting the conclusions of this study are available within the article and its supplementary information. All databases used in this study are publicly available. For identifying protein sequences and domain architecture, the Universal Protein Resource (https://www.uniprot.org/) was used. For identification of linear motifs within cell adhesion molecule intracellular domains, the Eukaryotic Linear Motif (ELM) resource (http://elm.eu.org/) was used. Additional microscopy replicates are available through Figshare at the following link: https://doi.org/10.6084/m9.figshare.21647546.v1. Source Data are provided with this paper.

Field-specific reporting

Please select the one below that is the best fit for your research. If you are not sure, read the appropriate sections before making your selection.

☒ Life sciences ☐ Behavioural & social sciences ☐ Ecological, evolutionary & environmental sciences

For a reference copy of the document with all sections, see nature.com/documents/nr-reporting-summary-flat.pdf

Life sciences study design

All studies must disclose on these points even when the disclosure is negative.

Sample size	No statistical tests were applied to determine sample size. Most sample sizes were kept large between 10-20 measurements with a coefficient of variation of approximately 25%. When experimental constraints limited sample size, a minimum of three independent replicates were measured and confirmed to be consistent.
Data exclusions	No data were excluded from the analysis. Microscopy images of wells containing clearly visible plastic contaminants or in which cells or spheroids were partially out of the field of view were not quantified.
Replication	Experiments are representative of at least three individual replicates with consistent results. Unless noted otherwise, replicates were combined for data analysis.
Randomization	Not relevant as covariate grouping was not applied.
Blinding	Data collection was automated and applied evenly between wells using a high content confocal microscope. For data analysis, blinding was applied during quantification in Fiji or Harmony software. Data analysis was automated using a macro for Fig. 2, Extended Data Fig. 4, Extended Data Fig. 6, Extended Data Fig. 10, Extended Data Fig. 12, Extended Data Fig. 15.

Reporting for specific materials, systems and methods

We require information from authors about some types of materials, experimental systems and methods used in many studies. Here, indicate whether each material, system or method listed is relevant to your study. If you are not sure if a list item applies to your research, read the appropriate section before selecting a response.

Materials & experimental systems

n/a	Involved in the study	
☐	☒	Antibodies
☐	☒	Eukaryotic cell lines
☒	☐	Palaeontology and archaeology
☐	☒	Animals and other organisms
☒	☐	Human research participants
☒	☐	Clinical data
☒	☐	Dual use research of concern

Methods

n/a	Involved in the study	
☒	☐	ChIP-seq
☐	☒	Flow cytometry
☒	☐	MRI-based neuroimaging

Antibodies

Antibodies used	1. DYKDDDDK Epitope Tag Alexa Fluor® 647-conjugated Antibody (clone 1042E) Rabbit R&D Systems (catalog #IC8529R) lot: AEOB0118081 Dilution: 1:100<br>2. DYKDDDDK Epitope Tag Alexa Fluor® 488-conjugated Antibody (clone 1042E) Rabbit R&D Systems (catalog #IC8529G) lot:

	AEOA0521031 Dilution 1:100<br>3. Myc-Tag (clone 9B11) Mouse mAb (AlexaFluor® 647 Conjugate) Cell signaling technology (catalog # 2233) lot: 25 Dilution 1:100<br>4. HA-Tag (6E2) Mouse mAb (AlexaFluor® 647 Conjugate) Cell signaling technology (catalog # 3444) lot: 15 Dilution 1:100<br>5. Human HGFR/c-MET(clone 95106) AlexaFluor® 488-conjugated Antibody R&D Systems (catalog# FAB3582G) lot: ADUM0117051 Dilution 1:50<br>6. EGFR Antibody (clone DH8.3) [AlexaFluor® 647] Novusbio (Catalog # 50599AF647) Dilution: 1:50<br>7. Anti- 6XHis tag (clone HIS.H8) antibody Abcam (Catalog #ab18184) Dilution 1:100
Validation	1. DYKDDDDK Epitope Tag Alexa Fluor® 647-conjugated Antibody was validated in HEK293 human embryonic kidney cell line transfected with DYKDDDDK-tagged proteins for flow cytometry by the manufacturer as reported on their website.<br>2. DYKDDDDK Epitope Tag Alexa Fluor® 488-conjugated Antibody was validated in HEK293 human embryonic kidney cell line transfected with DYKDDDDK-tagged proteins for flow cytometry by the manufacturer as reported on their website.<br>3. Myc-Tag (clone 9B11) Mouse mAb (AlexaFluor® 647 Conjugate) was validated by flow cytometric analysis of COS cells (fibroblast-like cell lines derived from monkey kidney tissue), transfected with Myc-tagged Akt by the manufacturer as reported on their website.<br>4. HA-Tag (6E2) Mouse mAb (AlexaFluor® 647 Conjugate) was Validated by flow cytometric analysis of COS cells transfected with HA-tagged DLL1 by the manufacturer as reported on their website.<br>5. Human HGFR/c-MET(clone 95106) AlexaFluor® 488-conjugated Antibody was validated by flow cytometry of MDA-MB-231 human breast cancer cell line by the manufacturer as reported on their website.<br>6. EGFR Antibody (clone DH8.3) [AlexaFluor® 647] was reported to be validated for flow cytometry on the manufacturer's website.<br>7. Anti- 6XHis tag (clone HIS.H8) antibody was validated by staining 6X His tag in transfected human HEK293 cells by Immunocytochemistry by the manufacturer as reported on their website.

Eukaryotic cell lines

Policy information about cell lines

Cell line source(s)	L929 mouse fibroblast cells (ATCC# CCL-1 were purchased from the American Type Culture Collection.<br>Madin- Darby Canine Kidney (MDCK) cells were a gift from the Mostov lab and originally sourced from Daniel Louvard at the European Molecular Biology Laboratory (Heidelberg, Germany)<br>Lenti-X™ 293T Cell Line was purchased from Takara Bio (Cat # 632180)<br>WA09 Human ES cells were purchased from WiCell (ID WAe009-A)
Authentication	Cell lines were authenticated by morphology and growth characteristics.
Mycoplasma contamination	Cell lines were confirmed to test negative for mycoplasma by the manufacturer
Commonly misidentified lines<br>(See ICLAC register)	No commonly misidentified cell lines were used.

Animals and other organisms

Policy information about studies involving animals; ARRIVE guidelines recommended for reporting animal research

Laboratory animals	Small intestinal crypts were dissociated from the duodenum of male C57BL/6 mice between 6-12 weeks of age
Wild animals	not applicable
Field-collected samples	not applicable
Ethics oversight	Mice were maintained in the University of California San Francisco (UCSF) specific pathogen-free animal facility. All maintenance and experiments were carried out in accordance with the guidelines established by the Institutional Animal Care and Use Committee and Laboratory Animal Resource Center. All experimental procedures were approved by the Laboratory Animal Resource Center at UCSF. Mice were housed in the UCSF LARC Animal Care Facilities at UCSF Parnassus. They were housed in an individual specific pathogen free suite. They were housed with up to 5 mice per cage in ventilator cages, with ad libitum food and water on a 12-hour light cycle and controlled temperature and humidity conditions (68-79 °F and 30– 70%).

Note that full information on the approval of the study protocol must also be provided in the manuscript.

Flow Cytometry

Plots

Confirm that:

☒ The axis labels state the marker and fluorochrome used (e.g. CD4-FITC).

☒ The axis scales are clearly visible. Include numbers along axes only for bottom left plot of group (a 'group' is an analysis of identical markers).

☒ All plots are contour plots with outliers or pseudocolor plots.

☒ A numerical value for number of cells or percentage (with statistics) is provided.

Methodology

Sample preparation	To confirm the expression level of synCAMs in each cell line, the cells were analyzed by FACS. The cells were detached with TrypLE and transferred to a round-bottom 96- well plate. The cells were pelleted by centrifugation (4 min, 400 g), the supernatant was removed, and the cells were resuspended in 40 uL PBS containing a fluorescent-dye conjugated antibody. Cells were stained for 50 minutes at 4°C. The cells were then washed twice with PBS and resuspended in PBS with 5% FBS. The cells were then analyzed by flow cytometry (BD LSR II).
Instrument	Cell sorting and flow cytometry was carried out using FACSAria II Cell Sorter or LSR II Flow Cytometer (Beckton-Dickinson).
Software	Data was analyzed with FlowJo™ (Mac v10.8.)
Cell population abundance	For flow cytometry histograms and pseudocolor plots, all events from sample are shown to enable direct comparison between samples.
Gating strategy	A gating strategy was not applied to the flow cytometry data in this study.

☒ Tick this box to confirm that a figure exemplifying the gating strategy is provided in the Supplementary Information.
